# Dynamic Assembly of Pentamer-Based Protein Nanotubes

**DOI:** 10.1021/acsnano.4c16192

**Published:** 2025-02-24

**Authors:** Lukasz Koziej, Farzad Fatehi, Marta Aleksejczuk, Matthew J. Byrne, Jonathan G. Heddle, Reidun Twarock, Yusuke Azuma

**Affiliations:** † Malopolska Centre of Biotechnology, Jagiellonian University, Krakow 30-387, Poland; ‡ Departments of Mathematics, University of York, York YO10 5DD, U.K.; § Astbury Centre for Structural Molecular Biology, University of Leeds, Leeds LS2 9JT, U.K.; ∥ School of Biological and Biomedical Sciences, Durham University, Durham DH1 3LE, U.K.; ⊥ Department of Biology, University of York, York YO10 5DD, U.K.

**Keywords:** protein cage, non-quasi-equivalent, geometry, cryo-EM, bionanotechnology

## Abstract

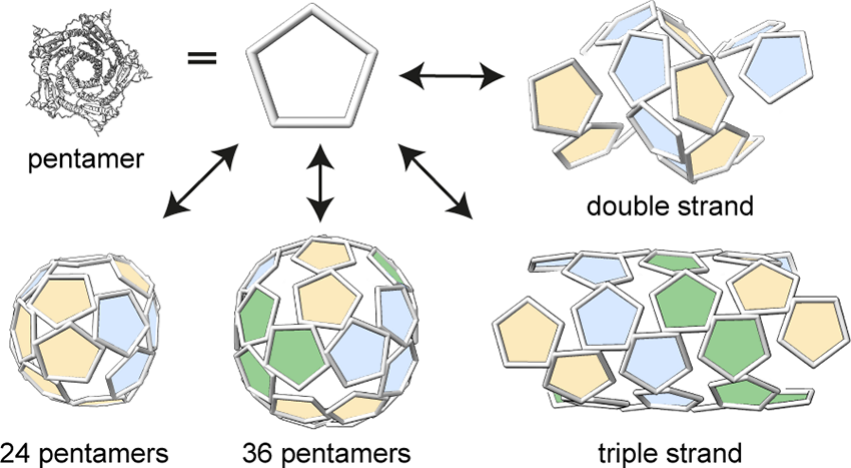

Hollow proteinaceous particles are useful nanometric containers for delivery and catalysis. Understanding the molecular mechanisms and the geometrical theory behind the polymorphic protein assemblies provides a basis for designing ones with the desired morphology. As such, we found that a circularly permuted variant of a cage-forming enzyme, *Aquifex aeolicus* lumazine synthase, cpAaLS, assembles into a variety of hollow spherical and cylindrical structures in response to changes in ionic strength. Cryogenic electron microscopy revealed that these structures are composed entirely of pentameric subunits, and the dramatic cage-to-tube transformation is attributed to the moderately hindered 3-fold symmetry interaction and the imparted torsion angle of the building blocks, where both mechanisms are mediated by an α-helix domain that is untethered from the native position by circular permutation. Mathematical modeling suggests that the unique double- and triple-stranded helical arrangements of subunits are optimal tiling patterns, while different geometries should be possible by modulating the interaction angles of the pentagons. These structural insights into dynamic, pentamer-based protein cages and nanotubes afford guidelines for designing nanoarchitectures with customized morphology and assembly characteristics.

Engineering of biomolecular assemblies with precisely defined structure and functionality is the ultimate goal of bionanotechnology. While nucleic acids are the favored materials in the field,^1^ substantial efforts have been directed to the design of nanoarchitectures based on protein building blocks.^2^ Hollow spherical or cylindrical structures, called protein cages^3−11^ or nanotubes,^12−15^ respectively, are particularly interesting in this context because of their prospective applications in delivery,^16−18^ catalysis,^19−21^ and nanomaterial construction.^22,23^ Naturally occurring protein assemblies, such as viral capsids, provide design concepts as well as reengineering platforms for customized nanodevice development.^24,25^

Many viral coat proteins can assemble into particles with a range of sizes and shapes when reconstituted in vitro.^26,27^ Geometrical patterns defined by quasi-equivalence (Casper-Klug) theory explain such polymorphic behavior.^28^ The variable number of hexamers filling in the gap between pentameric subunits at the vertices results in cage expansions and irregular forms.^29,30^ Such a dynamic, polymorphic feature is useful for customizing assemblies,^27,31^ as demonstrated with the cowpea chlorotic mottle virus (CCMV) coat protein, which assembles around DNA origami structures and protects them from degradation.^32^

Unique polymorphic behavior has been observed for engineered variants of *Aquifex aeolicus* lumazine synthase.^33,34^ While the wild-type protein, AaLS-wt, self-assembles into a ∼ 16 nm dodecahedral structure composed of 60 identical monomers (Figure 1A),^35^ the negatively supercharged variants AaLS-neg and AaLS-13 adopt expanded 360- and 720-mer assemblies, respectively, constructed entirely from pentameric capsomers.^36−38^ Moreover, a circularly permuted variant of AaLS, cpAaLS(119) (Figure 1B), forms not only ∼24 nm and ∼28 nm expanded spherical cages, but also straight ∼24 nm-wide tubes of variable length.^39^ Although such characteristics potentially present novel design principles,^31^ the geometric blueprints and the molecular mechanisms underlying this polymorphic behavior have remained unknown. Using cryo-EM and mathematical modeling, we elucidated how pentameric building blocks can controllably and dynamically assemble into specific spherical and tubular structures in preference to the other possible particle morphologies.

**Figure 1:**
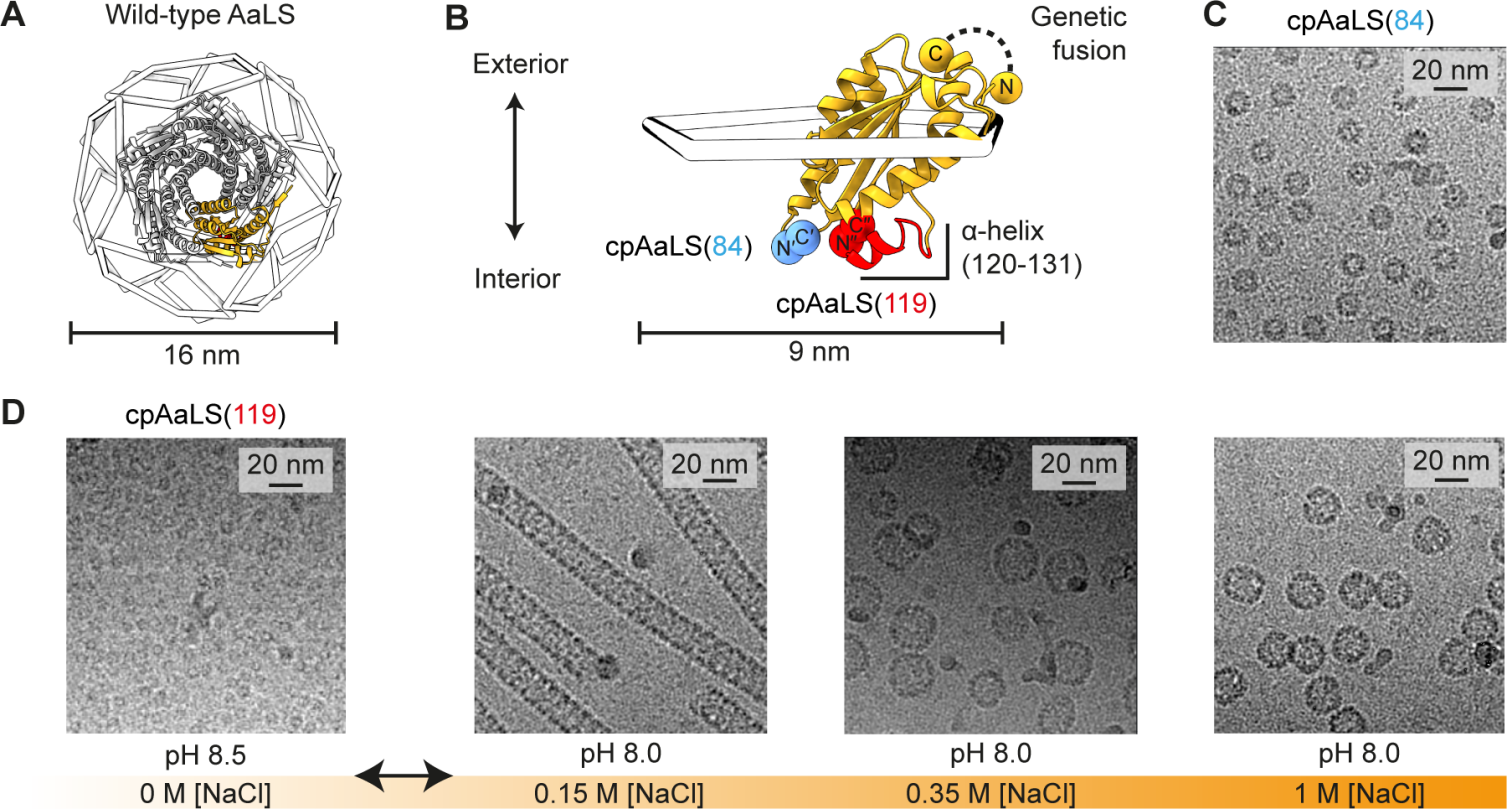
Assembly control of circularly permuted AaLS. (A) Structure of the dodecahedral wild-type AaLS cage (PDB ID 1HQK), shown as 12 wire pentagons with a ribbon diagram of a representative pentamer (gray) and protomer (orange). (B) Design of the circularly permuted variants, cpAaLS(84) and cpAaLS(119). The peptide linker connecting the native *N*- and C- termini (GTGGSGSS) is shown as a black dashed line. The new termini, C′(84) and N′(85) (blue) or C″(119) and N″(120) (red), are indicated by spheres. The α-helix(120–131), untethered by circular permutation for cpAaLS(119), is highlighted in red. (C,D) Cryo-EM micrographs of the cpAaLS(84) cage (C) and the NaCl- and pH-dependent cpAaLS(119) assemblies (D).

## Results and Discussion

### Salt- and pH-Dependent Assembly of cpAaLS(119)

We previously designed two circularly permuted variants of AaLS and confirmed that their morphologies depend on the positions of the newly generated *N*- and C-termini.^39−41^ The native terminal amino acids were connected via an octapeptide linker using genetic fusion and new sequence termini were introduced either between residues 84 and 85 or 119 and 120, yielding cpAaLS(84) or cpAaLS(119), respectively (Figure 1B). While cpAaLS(119) exhibits polymorphic behavior as discussed above, the cpAaLS(84) protein forms a ∼16 nm diameter homogeneous cage structure, similar to AaLS-wt, and serves as a control for the experiments described below (Figure 1C).

While characterizing the cpAaLS(119) variant, we unexpectedly found that the cage-like structures disassemble into fragments at low ionic strength and alkaline conditions. The protein was heterologously produced in *Escherichia coli* and isolated using ion exchange chromatography. Subsequent buffer exchange to 5 mM Tris-HCl buffer at pH 8.5 resulted in almost completely disassembled fragments, confirmed by size-exclusion chromatography coupled with right/low-angle light scattering detectors (SEC-RALS/LALS) (Figure S1).

Isolation of the cpAaLS(119) capsomers enabled systematic investigation of the reassembly process. Cage fragments were subjected to a rapid buffer exchange to 50 mM Tris-HCl buffer at pH 8.0 containing varying concentrations of NaCl (Figure S2A), and the resulting assemblies were analyzed by SEC and cryo-electron microscopy (cryo-EM) (Figures 1D and S2B–E). While remaining as unassembled fragments in the absence of salt (Figure S2B), adding 0.15 M NaCl facilitated cpAaLS(119) tubular formation with ∼92% yield (Figure S2C). Further increasing NaCl concentration to 0.35 M yielded a mixture of the tubes and ∼28 nm spherical cages ∼44% and ∼46%, respectively (Figure S2D). In 1 M NaCl, the tubes remained a 2% minority, while the ∼28 nm cage constituted ∼30% and the smaller ∼24 nm cages dominated at ∼59% (Figure S2E). These results demonstrated that cpAaLS(119) assembly can be controlled by adjusting the ionic strength of the solution. As NaCl concentration increases, unassembled fragments are preferentially transformed into 24 nm wide tubes, as well as 28 nm-, and 24 nm spheres.

Small changes in buffer pH can modulate the salt-dependent assembly of cpAaLS(119). Lowering pH tends to favor the assemblies that appear at high ionic strength and *vice versa*. For example, with 1 M NaCl at pH 8.5, 8.0, and 7.5, the proportion of 24 nm spherical cages was 9%, 59%, and 74%, respectively (Figure S2E). Furthermore, salt/pH-dependent cpAaLS(119) cage assembly is fully reversible. When the tube and spherical cages were subjected to buffer exchange into 50 mM Tris-HCl buffer at pH 8.5, the protein was completely converted into disassembled cage fragments (Figure S3).

To test if the cpAaLS variants retained the extreme thermal stability from the parent AaLS-wt, which has a melting temperature (*T*_m_) greater than 120 °C,^35,42^ we performed a thermal shift assay based on tryptophan fluorescence, coupled with dynamic light scattering, at different pH and NaCl concentrations (Figure S4A). Irrespective of the tested buffer conditions, cpAaLS(84) did not exhibit substantial fluorescent changes up to 110 °C, indicating little protein unfolding (Figure S4B). Temperature-dependent protein denaturation was observed for cpAaLS(119) with a *T*_m_ of 88–103 °C, where higher ionic strength and lower pH tend to increase the thermal stability. This variant also showed a decrease in size, probably due to partial fragmentation, at 70–80 °C under conditions that promote tube formation (Figure S4C, pH 7.5 and 0.15 M NaCl). Topological and/or morphological alteration likely leads to a relatively lower heat tolerance of cpAaLS(119) than that of AaLS-wt.

### Geometric Blueprints of the cpAaLS Assemblies

**Figure 2:**
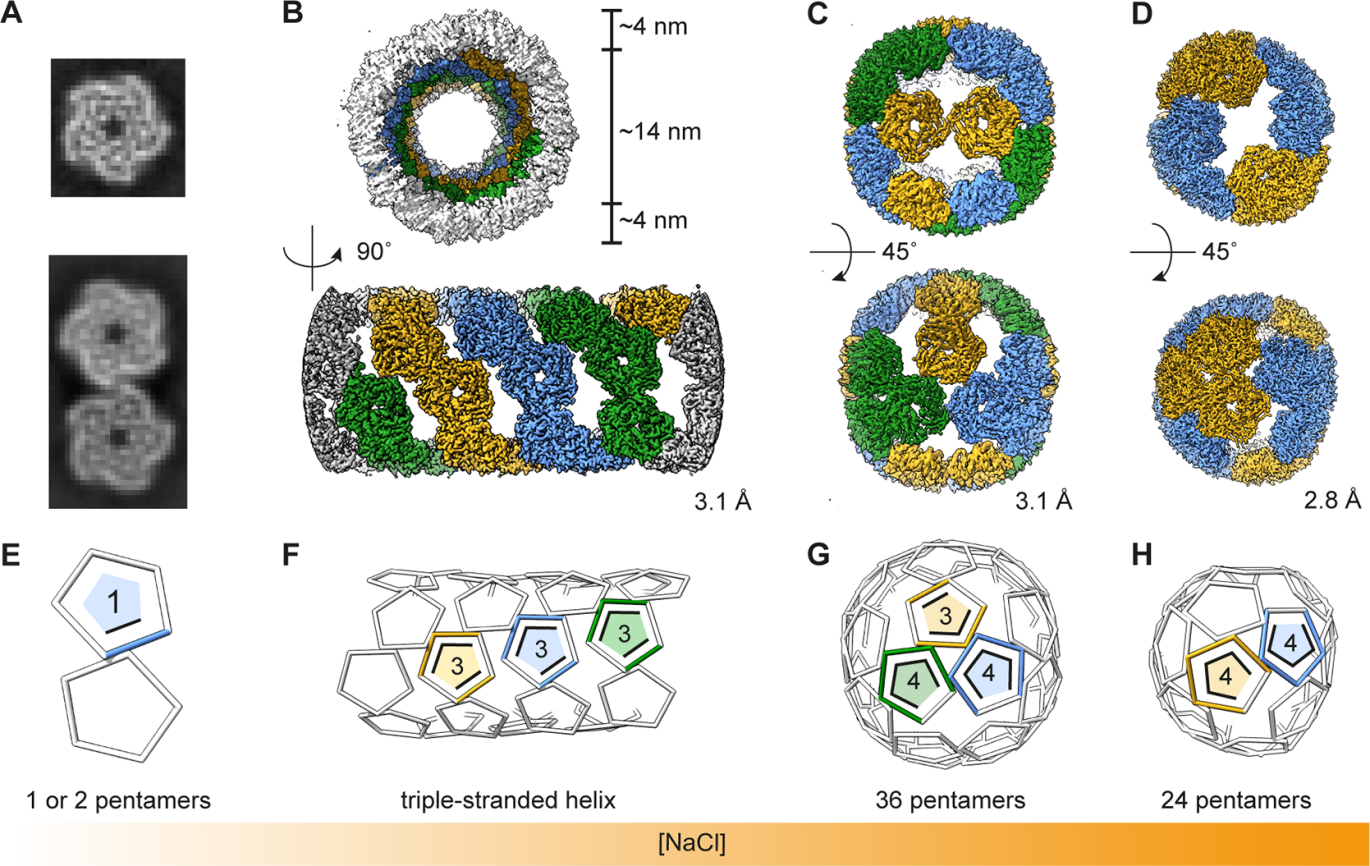
Cryo-EM structures of the cpAaLS(119) assemblies. (A–D) 2D classes (A) and 3D maps (B–D) of the cpAaLS assemblies, where colors (blue, orange, or green) indicate individual threads in the helical structure (B) or symmetry-related pentameric subunits in the spherical cages (C,D). The resolution of the final 3D reconstructions (GS-FSC at 0.143 cutoff) is provided at the right corner of each map. (E–H) The corresponding wire representation of the cpAaLS assemblies with the number of contacts per each asymmetric pentamer. Images are not to scale.

Control over cpAaLS(119) assembly by salt and pH allowed us to determine the structures of individual morphologies using cryo-EM single particle and helical reconstruction (Figures 2, S5, and S6). As previously hypothesized,^39^ all the structures were found to consist exclusively of the pentameric building blocks. The cpAaLS(84) assembly resembles the AaLS-wt cage, where each pentamer interacts with five neighboring subunits in a dodecahedral arrangement (Figure S5A). In contrast, cpAaLS(119) assemblies are non-quasi-equivalent, where at least one interface of the pentameric subunits remains uncontacted. The 24 and 28 nm spherical cages are composed of 24- and 36-pentamers, respectively, and both have tetrahedral symmetry (Figure 2C,D), resembling those formed by the previously engineered AaLS variants, NC-1 and AaLS-neg.^38,43^ In the tubular structure, the pentameric building blocks are arranged as a triple-stranded helix (Figure 2B), which is unique and has never been seen for any natural or engineered proteins.

The transformation of cpAaLS assemblies is accompanied by changes in the number of connections between the constituent pentamers. While the number of pentamer–pentamer contacts remains 0 or 1 without NaCl at pH 8.5, judged by the 2D-averaged cryo-EM images (Figure 2A,E), the mean contact number per pentamer increases to 3, 3.7, and 4 in the tubular, 28 nm-, and 24 nm spherical cages, respectively (Figure 2F–H). This trend suggests that increasing salt and/or lowering pH stabilizes interpentamer interactions.

AaLS is known to have a higher number of ionic interactions and hydrogen bonds at the pentamer–pentamer interface compared to an analogous lumazine synthase derived from *Bacillus subtilis*.^35^ These charge-driven interactions have been hypothesized to contribute to its extreme thermal stability. However, the salt-dependent increase in the number of contacts observed for cpAaLS(119) structures suggests that hydrophobic interactions are the major driving force for their assembly. Meanwhile, the pH-dependency can be explained by the net negative charge of the AaLS protein, which has a theoretical isoelectric point of 5.8. An increase in pH potentially endows the pentamers with increased negative surface charge, weakening interpentameric interactions due to charge repulsion.

### Molecular Mechanisms Underlying the Polymorphic Behavior

**Figure 3:**
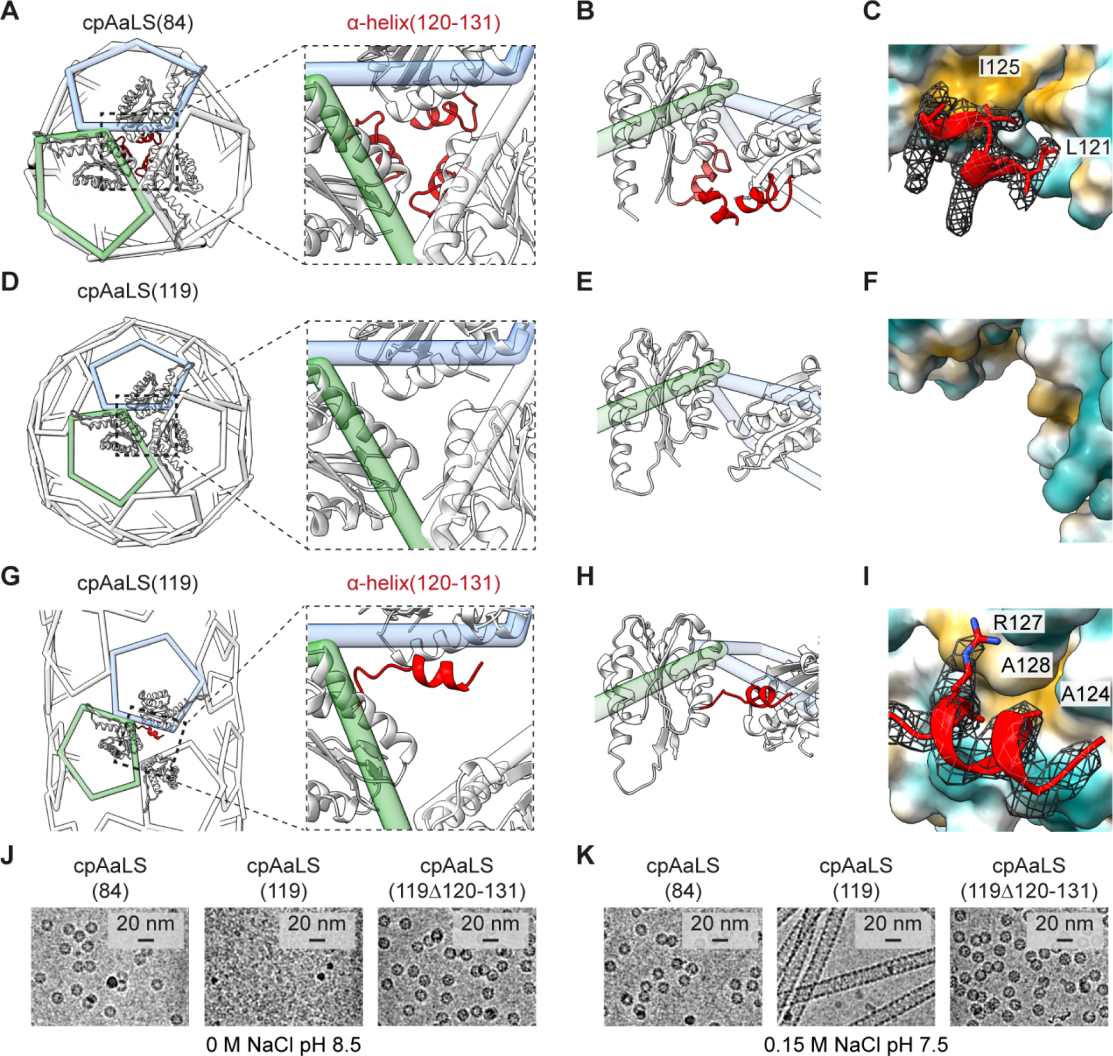
“Untethered” α-helix(120–131) facilitating the dynamic assembly of cpAaLS(119). (A) Wire diagram of the cpAaLS(84) assembly with an enlarged view of the 3-fold symmetry region. Three interacting monomers are shown as a ribbon with α-helix(120–131) highlighted in red. (B) Rotated side view of a pentamer pair (green and blue wire). The α-helix(120–131) domain from another monomer at the front is also shown to present the interaction at the 3-fold symmetry region in the cpAaLS(84) cage lumen. (C) Atomic interaction mode of the α-helix(120–131) domain in the cpAaLS(84) assembly. A unit is shown as a ribbon with amino acid side chains at the interface with the corresponding cryo-EM density map (mesh), and the interacting partners as hydrophilic (cyan) and hydrophobic (light brown) surfaces. (D–I) The corresponding representations for the cpAaLS(119) 24-pentameric spherical cage (D–F) and the tubular assembly (G–I), where the α-helix(120–131) is structurally disordered and was not modeled (D–F), or flipped to interact with an alternative surface (G–I), respectively. Panel (F) shows the same region as (C) to present the lack of cryoEM density corresponding to the α-helix(120–131) region. (J,K) Cryo-EM images of the cpAaLS(119) variant lacking α-helix(120–131), cpAaLS(119Δ120–131), compared to those of cpAaLS(84) and cpAaLS(119).

The atomic model of the cpAaLS(84) cage revealed that all the constituent pentamers interact with each other in the same manner as in the AaLS-wt assembly (Figure 3A). The 2-fold symmetry interface consists of a hydrophobic patch (L8, L141, and W137) surrounded by a hydrogen bond (H41) and ionic interactions (e.g., R40 and E5) (Figure S7A,B) while an α-helix region (120–131) forms 3-fold symmetric hydrophobic clusters (I121 and I125) in the cage interior (Figure 3B,C).

The bonding network observed at the 2-fold symmetry interface of AaLS-wt is approximately preserved for all the interacting pentamers in the cpAaLS(119) assemblies (Figure S7C–F). There is no swapping in the amino acid interaction partners in this region upon circular permutation and morphology change. In marked contrast, the (pseudo) 3-fold symmetrical interaction interface of cpAaLS(119) assemblies lacks cryoEM density corresponding to the N-terminal α-helix(120–131) (Figure 3D–F). This finding suggests that the α-helix is unable to form the native-like hydrophobic cluster, likely due to the disconnection of residues 119 and 120 for circular permutation (Figure 1B).

In the cpAaLS(119) tubes, the “untethered” α-helix(120–131) binds to the adjacent pentamer surface, which is a solvent-exposed region in wild-type-like assemblies (Figure 3G–I). Because of this non-native interaction, the α-helix(120–131) appears to block 3-fold symmetrical pentamer–pentamer interactions, which explains the uncontacted interfaces observed in the cpAaLS(119) assemblies (Figure 2E–H). Indeed, the deletion variant lacking the α-helix(120–131) domain, cpAaLS(119Δ120–131), does not exhibit polymorphic behavior, but assembles into only wild-type-like ∼16 nm spherical cages (Figure 3J,K). These results prove that the untethered α-helix(120–131) domain is essential for the non-native, expanded cage formation of cpAaLS(119).

In thermal shift assays, the cpAaLS(119Δ120–131) cage showed a similar behavior to cpAaLS(84): having no denaturation up to 110 °C (Figure S4). These results suggest that the reduced thermal stability of cpAaLS(119) is mainly due to the morphology, in which constituent pentamers lack one or two interactions with their neighbors, rather than the loss of the hydrophobic core in the region of the 3-fold symmetry axis.

The binding of the untethered α-helix(120–131) to neighboring pentamers appears to be weak and occasional. This is suggested by an atomic-level interaction mode in which an arginine (R127) and two alanine (A128 and A124) residues from the helical domain contact a serine in the linker connecting the native termini and a hydrophobic cleft formed between intrapentameric monomers, respectively (Figure 3I). Furthermore, substantial cryo-EM density corresponding to the α-helix(120–131) was found only for 2 protomers in each pentamer constituting the tubular assembly, and invisible in the spherical assemblies. The dynamic nature of the binding, which partially blocks pentamer–pentamer interactions, likely reflects multiple assembly states of cpAaLS(119).

### Capsomer Interaction Angles Facilitating Tube Formation

**Figure 4:**
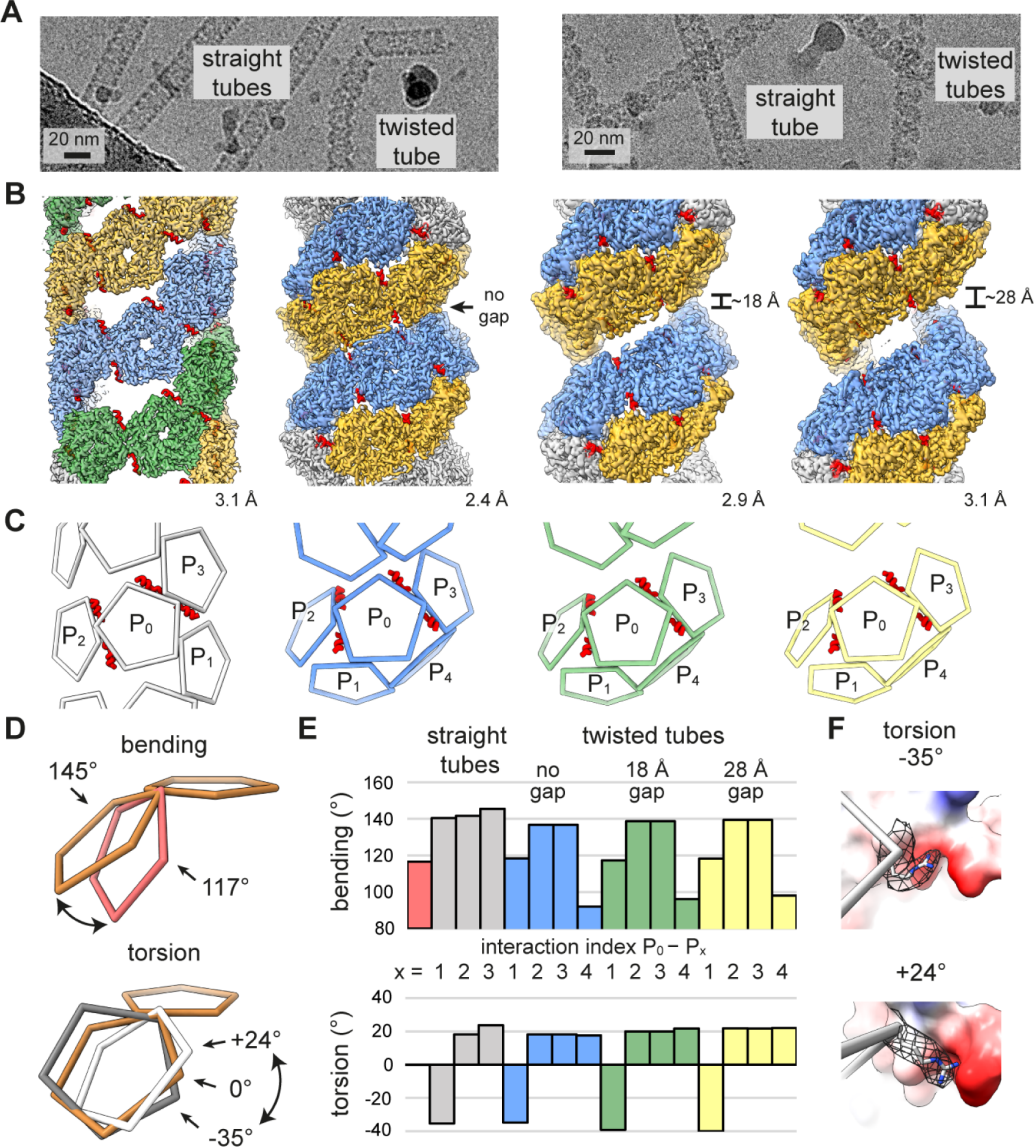
Capsomer interaction angles in the cpAaLS(119) tubes. (A) Cryo-EM micrographs of cpAaLS(119) (left) and cpAaLS(119, C37S, A85C) (right). (B) Cryo-EM maps of the straight tube composed of 3 evenly spaced helical strips (green, orange, and blue) and the twisted tubes featuring a variable gap (0, ∼18, or ∼28 Å) between dual strips (blue and orange). The resolution of the final 3D reconstructions (GS-FSC at 0.143 cutoff) is shown at the right corner of each map. The maps are not to scale. (C) The corresponding wire representations showing interactions of a pentamer (P_0_) with neighbors (P_1–4_). The α-helix(120–131) domains that accompany the intrathread interactions (P_0_–P_2/3_), otherwise invisible, are highlighted as red ribbons. (D,E) Conceptual representation (D) and the measured values (E) of the bending (top) and the torsion angles (bottom) between two pentamers. The wire diagrams show a pentamer–pentamer interaction in the cpAaLS(84) cage (red, top) and the cpAaLS(119) straight tubes (white and gray, bottom) with a reference having 0° torsion and 145° bending angles (brown). The interactions in the bar graph are colored and indexed as in (D), with those of the cpAaLS(84) cage (red bar) shown for comparison. (F) Two edges between interacting pentamers with the highest and the lowest torsion angles (−35° top, 24° bottom) observed in the cpAaLS(119) straight tube. The arginine side chain at the right vertex (R40, shown as gray sticks with mesh for the corresponding cryo-EM map) is flipped to maintain its interaction with a negatively charged patch (red surface) in the opposite pentamer.

The unique triple helix is not the only tubular structure formed by cpAaLS(119). In the cryo-EM micrographs, short and bent caterpillar-like objects, referred to as “twisted tubes” were also observed albeit at a very low frequency (Figure4A, left). Due to the limited number of particles, helical reconstruction was not initially possible. However, introduction of C37S and A85C mutations into cpAaLS(119) to give cpAaLS(119, C37S, A85C) and a modified assembly protocol (Figure S8) surreptitiously resulted in enrichment of the twisted tubular structure and allowed subsequent cryo-EM analysis (Figure 4A, right). Since the twisted tubular assemblies were heterogeneous, we divided particle images into 3 classes and determined the structures individually (Figure S9).

All three distinct twisted tube structures are composed of two compacted helical threads with 0-, 18-, and 28-Å gaps between them (Figure 4B). This spring-like arrangement rationalizes the bending tendency. We also realized that one of the four pentamer interactions in the twisted tube has an acute bending angle (Figure 4C, P_0_-P_4_ interaction). A possible disulfide bridge between two of the introduced cysteine residues at position 85 seems to support the unusual interaction mode, enhancing formation of the twisted tube (Figure S10).

As highlighted by the twisted tubes, the capsomer’s interaction angles and the entire morphology are interlinked. While each pentamer–pentamer interface in the regular AaLS-wt assembly is tightly fixed in a single pattern by the 2-fold and two 3-fold symmetry interactions, removal or modulation of these contacts by reengineering likely leads to a flexible connection and expanded cage-like structures. To analyze the relative positioning of the pentamers in the cpAaLS assemblies systematically, we built a script that calculates the bending and torsion angles of two interacting polygon-shaped multimers (Figure 4D). We named the computational angle generator “AngelaR”.

Analysis of the cpAaLS spherical assemblies using AngelaR showed that an increase in the bending angles between interacting pentamers results in larger-sized structures, as reported previously (Figure S11).^38^ In the twisted tubes, the increased gap between the compacted helical threads is accompanied by decreased bending and an increased torsion angle, a tendency agreeing with computational simulation using a pentagon-based helical model (Figure S12). In both the straight and twisted tubes, the pentamer–pentamer interaction adopts a wide range of torsion angles: −35°, 18°, and 24° for the straight tube, for example. The prominent negative torsion angles enable interthread dockings in these helical structures (Figure 4C,E, P_0_-P_1_ interaction).

AaLS proteins accept variable interaction angles using the flexible nature of the amino acid side chain. At the 2-fold symmetry interfaces of the cpAaLS(119) straight tube, for instance, an arginine residue (R40) is flipped to retain the interaction with the same negatively charged surface of the neighboring pentamer (Figure 4F), being accompanied by the interaction torsion-angle change.

When focusing on the individual thread of the tubular assemblies, the constituent pentamers are connected through consistently positive torsion angles (Figure 4C,E, P_0_-P_2/3_ interactions). This is required for helical arrangements of this type of pentameric building block, and the torsion angle defines the helical rise. If the torsion angle is zero degrees, as in the case of AaLS-wt assembly, the pentamers can form only a ring-shaped arrangement with no helical rise (Figure 4E, red bar). Notably, these intrathread capsomer interactions involve a pair of the untethered α-helices(120–131) that bind with a neighbor on the flipped position (Figure 4B,C, P_0_-P_2/3_ interactions), probably supporting their torsion angles in a certain range.

The α-helix(120–131) domain is structurally disordered and unseen in cryo-EM analysis of the cpAaLS(119) spherical cages (Figure 3D–F). Tubular assemblies have never been observed for other AaLS variants in which the α-helix(120–131) is tethered in the native position or removed (Figure 3J,K).^38,43−45^ Considering these observations together, we conclude that the untethered α-helix(120–131), which blocks the 3-fold symmetry interaction (Figure 3G) while imparting a consistent torsion angle to the subunits (Figure 4D,F, P_0_-P_2/3_ interactions), is the essential element for the transformation of the AaLS assembly from spherical to tubular structures.

### Mathematical Rationale for the Pentagon-Based Tubular Structures

**Figure 5:**
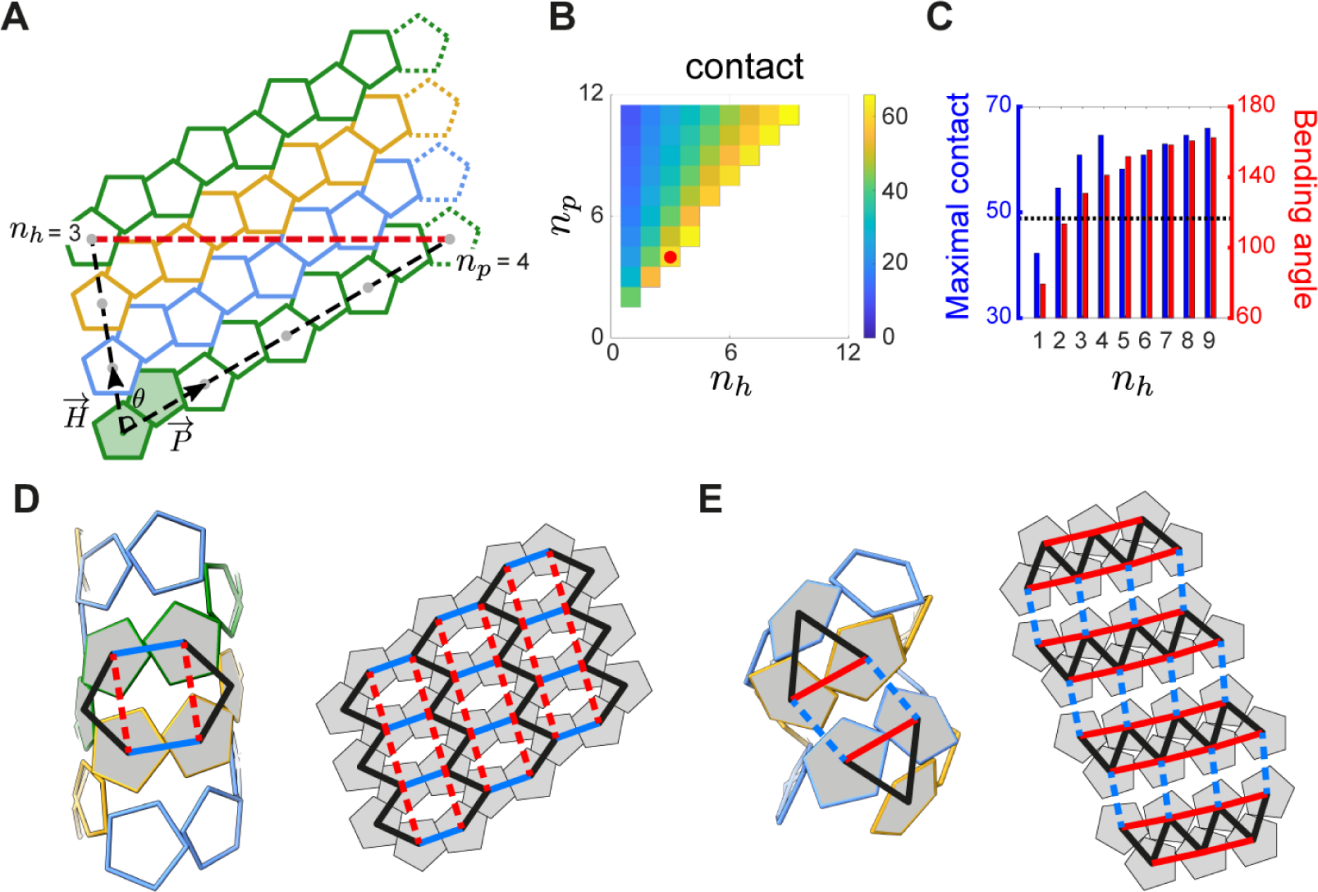
Geometric rationale for the cpAaLS(119) tubular assembly. (A) A tiling representation of the cpAaLS(119) straight tube composed of three pentamer strips (green, blue, and orange). The lattice model is formed from periodic repeats of pentamer pairs (shaded in green) with vectors pointing along (*P⃗*) and across (*H⃗*) helical threads at an angle (θ). The tubular architecture is defined by the translation steps, helicity (*n*_*h*_) and periodicity (*n*_*p*_) numbers, for two pentamers distanced by a single helical turn (gray dots connected by a black dashed line). The pentamers connected by the red dashed line are identical in the 3D tubular structure. (B) Heatmap presenting the percentage contact area between pentagonal edges in the tubular model for different (*n*_*h*_,*n*_*p*_) combinations. The experimentally observed (*n*_*h*_,*n*_*p*_) = (3,4) is indicated by a red dot. The structures in the blank area are geometrically or biologically forbidden. (C) The maximally possible contact area (blue) and the bending angle (red) for given *n*_*h*_ over all the possible choices of *n*_*p*_. The bending angle for the wild-type-like cpAaLS(84) cage is shown as a dotted line. (D,E) The interaction network rewiring between pentamers (shaded in gray) in the straight (D) and twisted tube (E), superimposed onto the 3D models (left) and the 2D tiling (right). Solid and dashed lines indicate contact and noncontact between pentamers, respectively. In the network transformation from the straight to the twisted tube, the blue contacts were lost, while the red ones were gained.

The spectrum of protein assemblies formed by a specific building block can be classified via tiling theory.^29,30,46−49^ The cpAaLS(119) tubes are very homogeneous in width, and no other structure with smaller and larger diameters was observed, indicating the building blocks adopt one specific geometry over others. To gain geometrical insight into pentamer-based tubular assemblies, we simulated all the possible helical arrangements formed by pentagons on a planar lattice (Figures 5A and S13). The model consists of a pair of pentamers (red) arranged with vectors pointing along (*P⃗*) and across (*H⃗*) helical threads, where the geometrically possible tubular structures are characterized by the number of distinct strips (helicity number *n*_*h*_) and the translation steps along *P⃗* (periodicity number *n*_*p*_). Some structures defined by these parameters, e.g. (*n*_*h*_,*n*_*p*_) = (4, 4), require flipped pentagons, which cannot be realized by asymmetric interaction surfaces of proteins (Figure S14). The cpAaLS(119) straight tubes adopt the tiling pattern with *n*_*h*_*= 3* and *n*_*p*_*= 4*, one of the smallest structures among all the geometrically and biologically possible options (Figure 5A,B), probably due to entropic preference.

The parameters (*n*_*h*_,*n*_*p*_)= (3, 4) appear to provide the optimal tiling pattern for the capsomer interaction of the AaLS protein. We next analyzed the percentage contact length between adjacent pentagons for different (*n*_*h*_,*n*_*p*_) combinations, finding that the maximal contact for each *n*_*h*_ is consistently obtained with the smallest possible *n*_*p*_ (Figure 5B). Comparison of the maximal contact for different *n*_*h*_ identifies (*n*_*h*_,*n*_*p*_) = (4, 5) as the local maximum, and (*n*_*h*_,*n*_*p*_) = (3, 4) as the second-best option among the smaller *n*_*h*_ values (Figure 5C, blue bars). We further benchmarked the bending angles between adjacent pentagons in the different geometric options (Figure 5C, red bars), indicating that the (*n*_*h*_,*n*_*p*_) = (3, 4) to be the smallest possible values larger than those of the wildtype particle (Figure 5C, dashed line). This angle is probably the most favored as it optimizes the contact surface of cpAaLS(119) pentamers. Meanwhile, these mathematical analyses suggest the possibility of other pentagon-based tubular assemblies by modulating the contact surface or bending angles between capsomers.

The seemingly distinct structure of the cpAaLS twisted tubes is geometrically related to that of the corresponding straight tubes. This is illustrated by the interaction network analysis that can predict alternatives by rewiring the connections of a given structure.^46^ Applying this approach, the cpAaLS straight tube features squashed hexagons drawn by connecting the centers of interacting pentamers (Figure 5D, black and blue solid lines). The only alternative to this can be obtained by deleting two existing contacts (blue solid lines) while generating two new ones (red dashed lines), yielding a triangle-based network that corresponds to the tiling blueprint of the twisted tubes (Figure 5E). This geometrical similarity could explain why a subtle change in the amino acid sequence and assembly condition resulted in the transformation between straight and twisted tubes. Furthermore, the network rewiring analysis implies that no other tiling patterns exist with this type of pentamer.

## Conclusion

Successful control over the assemblies and their near-atomic resolution structures revealed the molecular origin of the dynamic, polymorphic nature of a circularly permuted cage-forming protein. A short peptide domain, which is untethered from the native position by topological rearrangement, inhibits the 3-fold symmetry interaction and holds the building blocks with a certain torsion angle, leading to the dramatic conversion of the wild-type dodecahedron to the previously unknown helical arrangements of pentameric subunits. Given that another cpAaLS variant, NC-4, adopts a quasi-equivalent assembly consisting of pentamers and hexamers,^43^ these results highlight the morphological plasticity of AaLS and circular permutation as a powerful approach for modulating or potentially customizing cage-like structures.

The specific tiling patterns observed for the tubular structures are the optimal blueprints for this particular pentamer. Meanwhile, geometrically feasible structures are more diverse, suggesting the potential existence of other types of assemblies formed by naturally occurring or engineered proteins. Notably, the mathematical approaches used in this study, AngelaR, the helix builder, the planar lattice model, and the interaction network analysis,^46^ are general and should be, therefore, useful for characterizing or predicting yet unknown polygon-based structures.

In nature, protein tubes are abundant and involved in a variety of biological processes. They include protective storage and injection of genomic materials [viral capsids and tails],^50,51^ bacterial motility [flagella],^52^ or scaffolding cell shape and serving as rails for molecular transport [microtubules].^53^ Their broad functionalities inspire the engineering of customizable alternatives for applications in delivery, catalysis, and nanomaterial motility.^16,54,55^ Modular and readily modifiable cpAaLS(119) tubes are themselves an attractive platform for the prospective development of biomimetic nanodevices. Furthermore, our molecular and mathematical insights into the unique pentamer-based nanoarchitectures afford guidelines for the design of protein nanotubes with customized morphology and assembly dynamics.

## Materials and Methods

### Materials

All chemicals and biochemicals were purchased from Sigma-Aldrich (Burlington, MA, USA), New England BioLabs (Ipswich, MA, USA), or Thermo Fisher Scientific (Waltham, MA USA). Oligonucleotides were synthesized by Sigma-Aldrich. *E. coli* strains BL21-Gold(DE3) and DH5α competent cells were purchased from Agilent (Santa Clara, CA, USA) and Thermo Fisher Scientific, respectively. The plasmid pMG_cpAaLS_L8(119)^39^ and pMG_cpAaLS_L8(84)^41^ were kindly provided by Prof. Donald Hilvert (ETH Zurich, Switzerland).

### Molecular Cloning

The primers and plasmids used in this study are listed in Tables S1 and S2, respectively. Plasmid pMG_cpAaLS_L8(119Δ120–131) was prepared from pMG_cpAaLS_L8(119) by cassette cloning via NdeI and XhoI sites. Plasmid pMG_cpAaLS_L8(119, C37S) was prepared from pMG_cpAaLS_L8(119) by site-directed mutagenesis. Plasmid pMG_cpAaLS_L8(119, C37S) was used to prepare pMG_cpAaLS_L8(119, C37S, A85C) by site-directed mutagenesis. *E. coli* strain DH5α was used as the host cells for every cloning step. Sequences of plasmids were confirmed by DNA Sanger sequencing performed by Eurofins Genomics Europe Sequencing GmbH (München, Germany).

### Protein Expression

All proteins were produced in *E. coli* strain BL21-Gold(DE3) transformed with pMG vectors. The cells were cultured at 37 °C and 220 rpm in 0.5 L of lysogeny broth, Miller formulation (LB) medium supplemented with 100 μg/mL ampicillin until the OD600 reached ∼0.6, at which point protein production was induced by the addition of 0.5 mM isopropyl β-_D_-1-thiogalactopyranoside (IPTG). After culturing at 25 °C and 180 rpm for 20 h, cells were harvested by centrifugation at 3,000 × g, 4 °C for 10 min and then stored at −20 °C until protein purification.

### Protein Purification

Cell pellets from 250 mL cultures were resuspended in 100 mL of lysis buffer (25 mM Tris-HCl buffer (pH 8.5) containing 200 mM NaCl, 1 mM EDTA, and 1.2 mM MgOAc) supplemented with lysozyme (0.5 mg/mL) and DNaseI (5 μg/mL). After cell lysis by sonication, the insoluble fraction was removed by centrifugation for 25 min at 9,500 × g and 25 °C. The supernatant was heated at 70 °C for 1 h while stirring, followed by removal of insoluble fractions by centrifugation for 25 min at 9,500 × g and 25 °C. The supernatant was then diluted in a 1:1 volume ratio with 50 mM Tris-HCl buffer (pH 8.5) and loaded onto anion exchange HiTrap Q HP columns (4× 5 mL, Cytiva). After washing with 50 mM Tris-HCl buffer (pH 8.5) containing 100 mM NaCl, protein was eluted using a 0.1–1 M NaCl gradient. Fractions at approximately 400 mM NaCl were pooled and NaCl concentration was reduced to <2 mM with 50 mM Tris-HCl buffer (pH 8.5) using an ultrafiltration centrifugal filter unit (30 MWCO, Amicon Ultra-15, Merck Millipore). The protein solution was loaded onto an anion exchange Mono Q 5/50 GL column (Cytiva). After washing with the 50 mM Tris-HCl buffer (pH 8.5) containing 100 mM NaCl, the protein sample was eluted with a 0–1 M NaCl gradient. Fractions at approximately 400 mM NaCl were pooled and NaCl concentration was reduced to <2 mM with 5 mM Tris-HCl buffer (pH 8.5) using ultrafiltration. The protein sample was concentrated to approximately 300 μM and subjected to size-exclusion chromatography (SEC) using a Superdex 200 increase 10/300 column (Cytiva) running with 5 mM Tris-HCl buffer (pH 8.5) at room temperature (RT) and a flow rate of 0.75 mL/min. Peak fractions were pooled and protein was concentrated to approximately 345 μM (with respect to monomer concentration unless specified hereinafter) and kept at RT until further experiments. Protein concentration was routinely determined by absorbance at 280 nm (ε_280_ = 13,980 M^–1^ cm^–1^). Protein purity at each purification step was confirmed by SDS-PAGE with Coomassie R350 staining (Figure S16). After the first ion-exchange chromatography, protein concentration was kept <5 mg/mL throughout the purification process to minimize protein aggregation.

### Molecular Mass Analysis of cpAaLS(119) Capsomers

Apparent molecular mass of cpAaLS(119) in 50 mM Tris-HCl buffer (pH 8.5) was estimated by SEC coupled with right- and low-angle light scattering (RALS/LALS) detections (OMNISEC REVEAL, Malvern Panalytical Ltd., Malvern, UK) as described previously.^11^ Protein sample was buffer exchanged with 50 mM Tris-HCl buffer (pH 8.5) to 345 μM using ultrafiltration and analyzed by SEC-RALS/LALS using a Superdex 200 increase 10/300 column running at RT and 0.5 mL/min. Data was analyzed using Omnisec software (v11.10.7248.3) and parameters as follows: d*n*/d*c* = 0.185; d*A*/d*C* = 0; second virial coefficient (A2) = 0; RI = 1.333, viscosity = 0.7148 mPa s; detector oven temperature = 25 °C. The system was calibrated with conalbumin (75 kDa, 345 μM, 500 μL) (Gel Filtration High Molecular Weight Calibration Kit, GE Healthcare) in 50 mM Tris-HCl buffer (pH 8.5). Parameters used for the standard were as follows: intrinsic viscosity (IV, dL/g) = 0; the ratio between weight- and number-averaged molecular weight (*M*_W_/*M*_N_) = 1; a = 0.7 (Mark–Houwink parameter a). Settings for calculation method used for assessing molecular weight included: calibration type–triple detection, analysis type–calculate sample concentration from d*n*/d*c*.

### Assembly of cpAaLS(119) Protein Cages

Protein sample in 5 mM Tris-HCl buffer (pH 8.5) was mixed in 1:1 volume ratio with 2× buffer, followed by ultrafiltration in a corresponding 1× buffer [50 mM Tris-HCl buffer (pH 7.5, 8.0, or 8.5) containing 0 mM, 150 mM, 350 mM, or 1 M NaCl]. Protein was concentrated to approximately 172 μM and kept at RT for approximately 16 h, and then subjected to SEC using a Superose 6 increase column (Cytiva) at RT and a flow rate of 1 mL/min. The chromatograms were analyzed with ChromLab software (Bio-Rad) (Figure S2B–E, left). Prior to SEC, each sample was analyzed by cryo-EM using a Glacios microscope (Thermo Fisher Scientific).

### Cryo-EM Grid Preparation

Approximately 3.5 μL of the sample was applied onto a freshly glow discharged TEM grid (Quantifoil R2/2, Cu 200 mesh) and plunge vitrified into liquid ethane by a Vitrobot Mark IV (Thermo Fisher Scientific). For cryo-EM with a Glacios microscope, the following vitrification parameters were used: humidity 95%, temperature 10 °C. blot total 1, wait time 30 s, and drain time 0 s. Blot force and blot time were adjusted depending on the ionic strength of the buffer; 0 mM NaCl: 7 and 7 s, 150–350 mM NaCl: 6 and 6 s, 1 M NaCl: 5 and 5 s, respectively. For cryo-EM measurement on a Titan Krios G3i microscope (Thermo Fisher Scientific), the following vitrification parameters were used: humidity 95%, temperature 10 °C. blot total 2, wait time 30 s, blot force 3, blot time 3 s, and drain time 0 s.

### Cryo-EM with Glacios Microscope

All the cryo-EM micrographs were collected at the Cryo-EM Centre of the National Synchrotron Radiation Centre SOLARIS (Krakow, Poland). The micrographs, typically 20 for each variant and condition, were acquired on a Glacios microscope (Thermo Fisher Scientific) fitted with a Falcon 4 detector operated at 200 kV accelerating voltage, magnification of ×150k, and corresponding pixel size of 0.96 Å/px. The collected micrographs were analyzed using Fiji and cryoSPARC v4.2.1.^56,57^

### Disassembly of cpAaLS(119) Protein Cages

Protein solution in 5 mM Tris-HCl buffer (pH 8.5) was mixed in 1:1 volume ratio with 2× buffer, followed by ultrafiltration in a corresponding 1× buffer [50 mM Tris-HCl buffer (pH 8.0) containing 0.15 or 1 M NaCl] (Figure S3A). Protein was concentrated to approximately 172 μM and kept at RT for approximately 16 h. The protein assemblies were analyzed and isolated by SEC using a Superose 6 increase 10/300 column in corresponding 1× buffer at RT and a flow rate of 1 mL/min. Peak fractions were pooled, and protein was concentrated to approximately 90 μM in 50 mM Tris-HCl buffer (pH 8.5). After approximately 1 h, the sample was reanalyzed by SEC using the same column and conditions, except for the eluent of 50 mM Tris-HCl buffer (pH 8.5).

### Thermal Stability of cpAaLS Assemblies

Protein sample in 5 mM Tris-HCl buffer (pH 8.5) was mixed in 1:1 volume ratio with 2× buffer, followed by ultrafiltration in a corresponding 1× buffer [50 mM Tris-HCl buffer (pH 7.5) containing 0.15 or 1 M NaCl; or 50 mM Tris-HCl buffer (pH 8.5) containing 0 or 1 M NaCl] (Figure S4A). Protein was concentrated to approximately 172 μM and kept at RT for approximately 16 h. The protein solution was diluted to 57 μM with the corresponding buffers and transferred to a Prometheus high sensitivity glass capillary sealed with a dedicated sealing paste (NanoTemper Technologies). Thermal shift assay with differential scanning fluorimetry (DSF) and dynamic light scattering (DLS) detections were performed using a Prometheus PANTA Instrument (NanoTemper Technologies) over a 25–110 °C temperature range with a 1 °C/min ramp. The measurements were triplicate. Data sets were analyzed and merged using the PR.PantaAnalysis software.

### Cryo-EM Single Particle Reconstruction of cpAaLS(119) Capsomers

Cryo-EM data were acquired on a Titan Krios G3i microscope operated at 300 kV accelerating voltage, magnification of ×105k, and pixel size of 0.86 Å/px. A K3 direct electron detector used for data collection was fitted with BioQuantum Imaging Filter (Gatan) using a 20 eV slit and operated in counting mode. Imaged areas were exposed to 40 e^–^/Å^2^ total dose (corresponding to ∼16 e^–^/px/s dose rate measured in vacuum). Forty-frame movie stacks were obtained using under-focus optical conditions with a defocus range of −2.1 to −0.9 and 0.3 μm steps. The collected data sets were analyzed using cryoSPARC v4.2.1. First, “Patch Motion Correction” and “Patch CTF Estimation” steps were performed. Next, a “Blob Picking” step resulted in 224767 particles, and subsequent 2D classification produced classes corresponding to flat pentamers and pentamer pairs. Due to the preferential orientation of particles in the grid holes, 3D reconstruction was unsuccessful.

### Cryo-EM Single Particle Reconstruction of cpAaLS Spherical Cages

The cpAaLS(84) 16 nm cages, cpAaLS(119) 24 or 36 nm spherical assemblies were isolated by SEC using a Superose 6 increase 10/300 column in 50 mM Tris-HCl buffer (pH 8.0) containing 150 mM, 500 mM or 350 mM NaCl. The peak fractions were concentrated by ultrafiltration to approximately 28 μM (or 56 μM for cpAaLS(119) 24 nm cages) in the corresponding buffer and used for vitrification. To reduce salt concentration, the sample with cpAaLS(119) 24-pentamer cage was subjected to a quick 2-fold dilution with 50 mM Tris-HCl buffer (pH 8.0) (final 250 mM NaCl) prior to vitrification. Cryo-EM data was collected on a Titan Krios microscope, as described above with minor modifications, and analyzed using RELION v3.1 using parameters shown in Figure S5.^58^ Briefly, after motion correction and CTF estimation, approximately 500 particles were picked manually, and used for 2D classification as well as the generation of preliminary classes for template picking. After ab initio reconstruction in C1 using picked particles, 3D classification and 3D refinements were performed using icosahedral (I) or tetrahedral (T) symmetries (Figure S5). The particle stacks were subjected to iterative per-particle defocus and global CTF refinements, followed by Bayesian polishing. Gold-standard Fourier shell correlation and local map resolutions were calculated with 0.143 FSC cutoff. Prior to model fitting, the combined half-maps were sharpened with DeepEMhancer.

### Cryo-EM Helical Reconstruction of cpAaLS(119) Straight Tube

As for spherical cages, the cpAaLS(119) straight tube fraction was isolated by SEC using a Superose 6 increase 10/300 column in 50 mM Tris-HCl buffer (pH 8.0) containing 150 mM NaCl and concentrated by ultrafiltration to approximately 28 μM. Cryo-EM data were collected on a Titan Krios microscope, as described above, using a magnification of ×81k and a pixel size of 1.1 Å/px. Forty-frame movie stacks were obtained using super-resolution mode (0.55 Å/px) and under-focus optical conditions with a defocus range of −3.0 to −0.9 and 0.3 μm steps. The collected data set was analyzed using helical reconstruction in RELION v3.1 using parameters shown in Figure S6.^59^ Briefly, after motion correction (2× bin) and CTF estimation, straight helical segments were manually picked by selecting start-end coordinates and subjected to 2D classification. A cylinder with a 210-Å outer diameter was generated using relion_helix_toolbox and used as an initial model for 3D classification. The final 168323 particle stacks were used for 3D helical reconstruction, followed by iterative per-particle defocus, global CTF refinements, and Bayesian polishing. Gold-standard Fourier shell correlation and local map resolutions were calculated with 0.143 FSC cutoff. Prior to model fitting, the combined half-maps were sharpened with DeepEMhancer.^60^

### Assembly of cpAaLS(119, C37S, A85C) Twisted Tube

The cpAaLS(119, C37S, A85C) variant in 5 mM Tris-HCl buffer (pH 8.5) was mixed with 50 mM Tris-HCl buffer (pH 7.0) followed by buffer exchange using ultrafiltration with the same buffer (Figure S7). Protein was concentrated to approximately 172 μM and kept at RT for approximately 16 h. The resulting solution was then mixed in 1:1 volume ratio with 50 mM Tris-HCl buffer (pH 8.5) containing 0.3 M NaCl, followed by ultrafiltration in 50 mM Tris-HCl buffer (pH 8.5) containing 0.15 M NaCl and 1 mM tris(2-carboxyethyl)phosphine (TCEP). Protein was concentrated to approximately 172 μM and kept at RT for approximately 16 h. The protein assembly was then analyzed and isolated by SEC using a Superose 6 increase 10/300 column in 50 mM Tris-HCl buffer (pH 8.5) containing 0.15 M NaCl and 1 mM TCEP at RT and a flow rate of 1 mL/min. Peak fractions corresponding to nanotubes were pooled, and protein was concentrated to approximately 57 μM for cryo-EM analysis. An analog experiment was performed with the parent cpAaLS(119) variant for comparison.

### Cryo-EM Helical Reconstruction of cpAaLS(119, C37S, A85C) Twisted Tube

Cryo-EM data was collected on a Titan Krios G3i, as described above, using a magnification of 105k and a corresponding pixel size of 0.846 Å/px. Movie stacks (40 frames) were obtained using under-focus optical conditions with a defocus range of −1.5 to −0.9 and 0.3 μm steps. The collected data set was analyzed using “Helical Reconstruction” in cryoSPARC v4.4.1.^57^ First, “Patch Motion Correction” and “Patch CTF Estimation” steps were performed. Next, approximately 500 particles were picked manually. The acquired particles were subjected to 2D classification and used in the generation of preliminary classes for the subsequent template picking using the filament tracing tool (Figure S8). Particles were extracted using 2× binning. Following 2D classification, a cylinder with a 200/130 Å outer/inner diameter was generated and used as an initial model for initial 3D “Helical Refinement”. Following “Heterogeneous Refinements”, the particles sets were split based on uniformity into three independent structures with 1) ∼ 24.5-, 2) ∼ 22-, or 3) ∼ 20-Å helical rise. The resulting structures correspond to “twisted tube” with 1) ∼ 28-Å, 2) 18-Å, or 3) no/0-Å gap between dual helical threads. Prior to final 3D helical refinements, the particles were unbinned and subjected to additional 2D classification resulting in corresponding 192310, 807640, or 208379 stacks. Furthermore, the particles were subjected to “Reference Based Motion Correction”. During final 3D “Helical Refinements”, the particles and micrographs were subjected to per-particle defocus, global CTF refinements, and Ewald Sphere correction to generate high-resolution maps. Gold-standard Fourier shell correlation and local map resolutions were calculated with 0.143 FSC cutoff. Prior to model fitting, the combined half-maps were sharpened with DeepEMhancer.^60^

### Molecular Modeling

The initial atomic model was sourced from a X-ray crystal structure (1.6-Å resolution) of wild-type AaLS (PDB: 1HQK).^35^ Following rigid body fitting using ChimeraX v1.7,^61^ and manual modification in Coot,^62^ coordinates were flexibly fit with Isolde.^100^ The models were real-space refined in Phenix v1.20.1-4487.^101^ The final coordinates were validated using MolProbity,^63^ and the model statistics are presented in Table S3. The cryo-EM maps and atomic models were displayed using ChimeraX.

### Estimation of the Bending and Torsion Angles

The normal vectors to the planes defined by two adjacent pentamers were computed. If these vectors are in a plane with a line connecting the centers of the pentamers, the torsion angle is zero and the bending angle is 180 minus the angle between two normal vectors, which corresponds to the bending angle at the interface of the pentamers. If the normal vectors do not form a plane with the line connecting the centers of the pentamers, the torsion angle corresponds to the angle between the normal vector before and after it has been rotated into that plane. The code implementing this procedure is available from GitHub: (https://github.com/MathematicalComputationalVirology/TubeModeler “Script_measuring_angles.py”).

### Simulation of the Single-Stranded Helix Model for Twisted Tubes

The helix model was constructed by applying bending and torsion angles iteratively starting with an initial pentamer. The incoming pentamer is located at a position on the interface specified by a free parameter in the code. At that point, the construction is fully determined. The code implementing this procedure is available from GitHub: (https://github.com/MathematicalComputationalVirology/TubeModeler “Twisted_Tube_3D.m”).

### Mathematical Characterization of the Straight Tubes

The mathematically viable straight tubes with helicity number *n*_*h*_ and periodicity number *n*_*p*_ are characterized based on the construction shown in Figure S13. Taking the center of the green pentagon as the origin (*O*), its vertices *P*_*i*_ correspond to the fifth roots of unity:
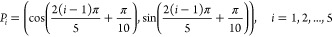


Then the vector *H⃗*= (a, b) is the translation vector between two strips. The vertices of the translated pentagon are Q*_i_* = *P*_*i*_ + *H⃗* for *i* = 1, 2,..., 5. Denoting by *R*_1_ the intersection point of the lines  and  and using coordinates values, one obtains:
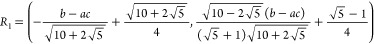
where
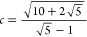


We choose the point  on *P*_3_*P*_4_ such that the length of  equals that of *R*_1_*P*_2_. As the contact pattern is the same along a strip, the length of  moreover equals that of *TR*_3_. Thus,  and *R*_3_ = *R*_1_ + (*P*_1_*– P*_3_). Since ,  is an anticlockwise rotation by 144° of *R*_1_ around the origin, i.e.,
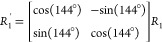


To obtain the helicity number *n*_*h*_ and periodicity number *n*_*p*_, the following equality should be satisfied for the second components of the vectors *H⃗* and *P⃗*:
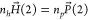


By substitution, the above equality then leads to the following relation:
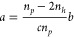


This equation demonstrates that the parameters *n*_*h*_ and *n*_*p*_ are geometrically linked.

### Procedure for Mathematical Model Building of Straight Tubes

The values of *b*, *n*_*p*_ and *n*_*h*_ define *a*, which gives vectors *H⃗* and *P⃗*. Starting with a pentagon, and using *b = 2.18* to match the biological structure, the coordinates of the pentagon are translated as shown in Figure S13. The contact area corresponds to the proportion of a pentagonal edge in contact with the edge of a neighboring pentamer, and is given as a percentage, i.e., . The code implementing this procedure is available from GitHub: (https://github.com/MathematicalComputationalVirology/TubeModeler “Straight_Tube_2D.m” and “Straight_Tube_3D.m”).

### Rewiring of the Capsomer Network^46^

The alternative architectures were obtained from a given structure with reference to its interaction network. In the case of the straight tubes, the interaction network is given by squashed hexagons, which are composed of two triangles and one square. Associating weights (including zero) to these edges, respecting (helical) symmetry-equivalent positions and connectivity, results in different structures. The only other option here is the twisted tube architecture.

## Data Availability

The code implementing the mathematical analysis is available from GitHub: https://github.com/MathematicalComputationalVirology/TubeModeler.

## Supporting Information Available

Experimental and data analysis schemes, mathematical models, protein and DNA primary sequences, and supporting data from size-exclusion chromatography coupled with right/low-angle light scattering (SEC-RALS/LALS), nano differential scanning fluorimetry (NanoDSF) coupled with dynamic light scattering (DLS), size-exclusion chromatography (SEC), cryo-electron microscopy (cryo-EM), sodium dodecyl sulfate gel electrophoresis (SDS-PAGE) (PDF)

## Accession Codes

Cryo-EM maps and atomic models have been deposited in the Electron Microscopy Data Bank (EMDB) and the Worldwide Protein Data Bank (wwPDB), respectively, with the following accession codes: EMDB-51006 and PDB 9G3P (12-pentamer cage), EMDB-51004 and PDB 9G3N (36-pentamer cage), EMDB-51003 and PDB 9G3M (straight tube), EMDB-51001 and PDB 9G3J (28-Å gap twisted tube), EMDB-51000 and PDB 9G3I (18-Å gap twisted tube), and EMDB-50999 and PDB 9G3H (0-Å gap twisted tube).

## Abbreviations

AaLS: *Aquifex aeolicus* lumazine synthase
cpAaLS: circularly permuted variants of AaLS
AaLS-wt: wildtype AaLS
cryo-EM: cryogenic electron microscopy
SEC: size-exclusion chromatography
SEC-RALS/LALS: SEC with right/low-angle light scattering detectors
*T*m: melting temperature at which point 50% of protein is unfolded
GS-FSC: gold-standard Fourier shell correlation
