# Supplementary material for: Dynamic Assembly of Pentamer-Based Protein Nanotubes
Source: ACS Nano. 2025 Feb 24;19(9):8786–98. doi: 10.1021/acsnano.4c16192 (PMC11912573; doi:10.1021/acsnano.4c16192)
Supplement: Supplementary file 1 — nn4c16192_si_001.pdf [file nn4c16192_si_001.pdf]

Supporting Information for

Dynamic assembly of pentamer-based protein nanotubes

*Lukasz Koziej[a], Farzad Fatehi[b], Marta Aleksejczuk[a], Matthew J. Byrne[c], Jonathan G. Heddle[a,d], Reidun Twarock[b,e], and Yusuke Azuma[a]**

[a] Malopolska Centre of Biotechnology, Jagiellonian University, Krakow 30-387, Poland

[b] Departments of Mathematics, University of York, York YO10 5DD, United Kingdom

[c] Astbury Centre for Structural Molecular Biology, University of Leeds, Leeds LS2 9JT, United Kingdom

[d] School of Biological and Biomedical Sciences, Durham University, Durham DH1 3LE, United Kingdom

[e] Department of Biology, University of York, York YO10 5DD, United Kingdom

*E-mail: yusuke.azuma@uj.edu.pl

Index

Figure S1. Disassembly of cpAaLS(119) cages in a low-ionic-strength buffer at alkaline pH.

Figure S2. Ionic strength and pH-dependent assembly of cpAaLS(119).

Figure S3. Salt and pH-dependent disassembly of cpAaLS(119) protein cages into capsomers.

Figure S4. Thermal stability of cpAaLS assemblies.

Figure S5. Single particle reconstruction of cpAaLS(84) and cpAaLS(119) spherical cages.

Figure S6. Helical reconstruction of cpAaLS(119) straight tube.

Figure S7. Bonding network between pentamers at the (pseudo) 2-fold symmetry in cpAaLS cages.

Figure S8. Assembly of cpAaLS(119, C37S, A85C) into twisted tubes.

Figure S9. Helical reconstruction of cpAaLS(119, C37S, A85C) twisted tube.

Figure S10. Potential disulfide bridge facilitating the acute bending angle in the pentamer-pentamer interaction.

Figure S11. Capsomer interaction angles in spherical cages.

Figure S12. Correlation between pentamer interaction angles and helical rise.

Figure S13. Mathematical characterization of the straight tubes.

Figure S14. Tiling pattern and resulting tubular structures.

Figure S15. Tiling pattern violating the biologically occurring interactions.

Figure S16. Primary sequence and purification of cpAaLS variants.

Table S1. Oligonucleotides used in this study.

Table S2. Plasmids used in this study.

Table S3. Cryo-EM data collection and structure refinement statistics.

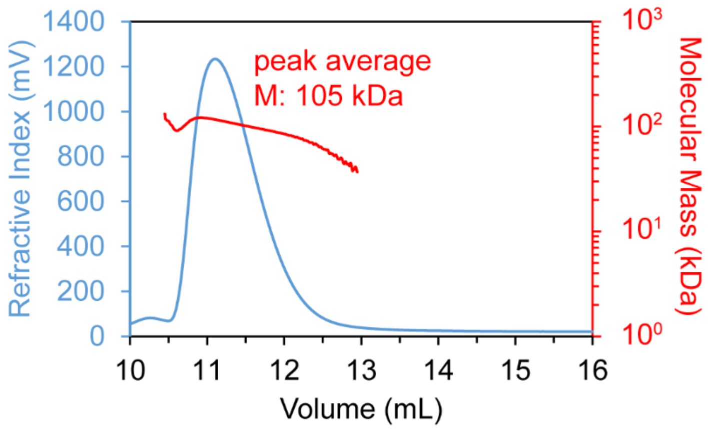

Figure S1. Disassembly of cpAaLS(119) cages in a low-ionic-strength buffer at alkaline pH.

The cpAaLS(119) protein was analyzed by size-exclusion chromatography coupled with right/low-angle light scattering detectors (SEC-RALS/LALS) in 50 mM Tris-HCl buffer at pH 8.5, estimating the average molecular mass (M) of 105.2 kDa. This likely corresponds to a mixture of cpAaLS pentamer (86.5 kDa) and its dimer (174.3 kDa), which was further confirmed by cryo-EM (Fig . 2A).

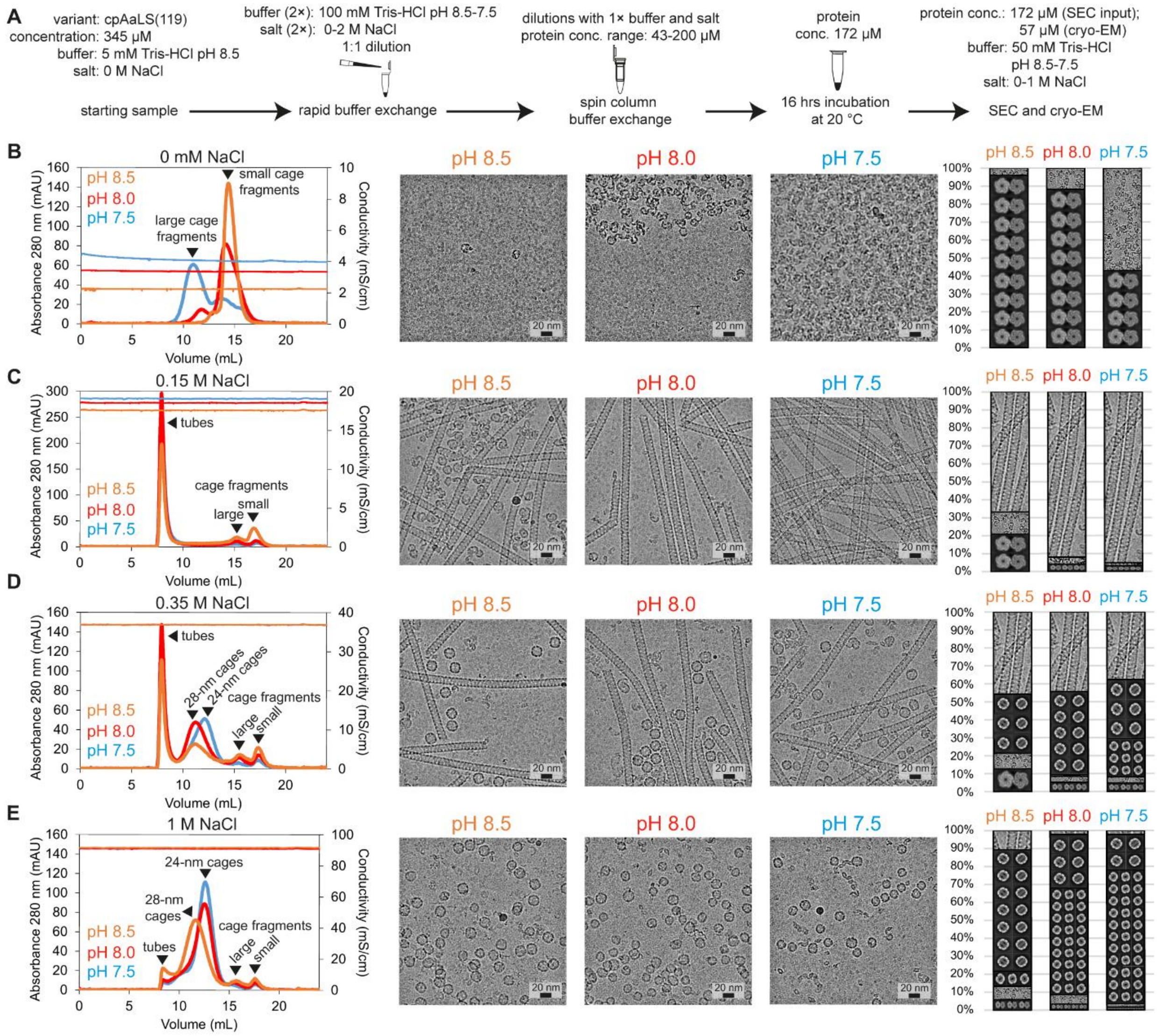

Figure S2. Ionic strength and pH-dependent assembly of cpAaLS(119).

(A) Experimental scheme to assemble cpAaLS(119) proteins under different buffer conditions. (B-E) Size-exclusion chromatography (SEC) profiles (left), representative cryo-EM micrographs (middle), and relative distribution of cpAaLS(119) assembly states (right) in a buffer containing 0 (B), 0.15 (C), 0.35 (D), or 1 M (E) NaCl at pH 8.5 (orange), 8.0 (red), or 7.5 (blue). The relative distribution of the assembly states was quantified by the integrating peak areas of the SEC traces. The 24-nm and 28-nm cages were discriminated by manual inspection and classification of spherical particles from ~20 micrographs. Small and large cage fragments in SEC profiles likely represent different oligomerization statuses of pentamers. In the SEC profiles, conductivities are also shown as a reference for the buffer ionic strength which is slightly dependent on pH due to the different ionization levels of Tris base.

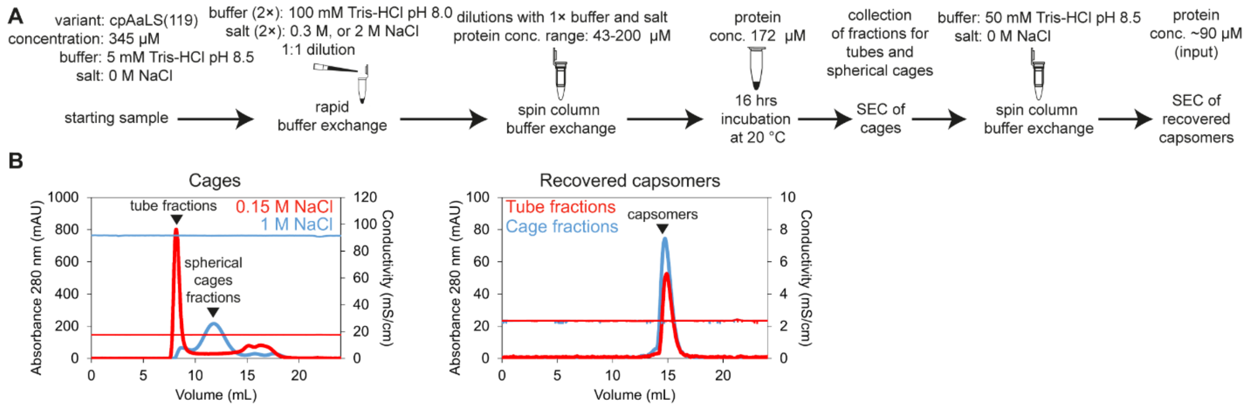

Figure S3. Salt and pH-dependent disassembly of cpAaLS(119) protein cages into capsomers.

(A) Experimental scheme describing stepwise cage assembly-disassembly by buffer exchange. (B) 50 mM Tris-HCl buffers (pH 8.0) containing 0.15 M (red) or 1 M (blue) NaCl were used for tubular and spherical cage formation, respectively, and the major assemblies were isolated by SEC (bottom left). Following buffer exchange to 50 mM Tris-HCl buffer (pH 8.5) resulted in nearly 100% cage disassembly into the fragments (capsomers, bottom right). Conductivities are shown as a reference for the buffer ionic strength.

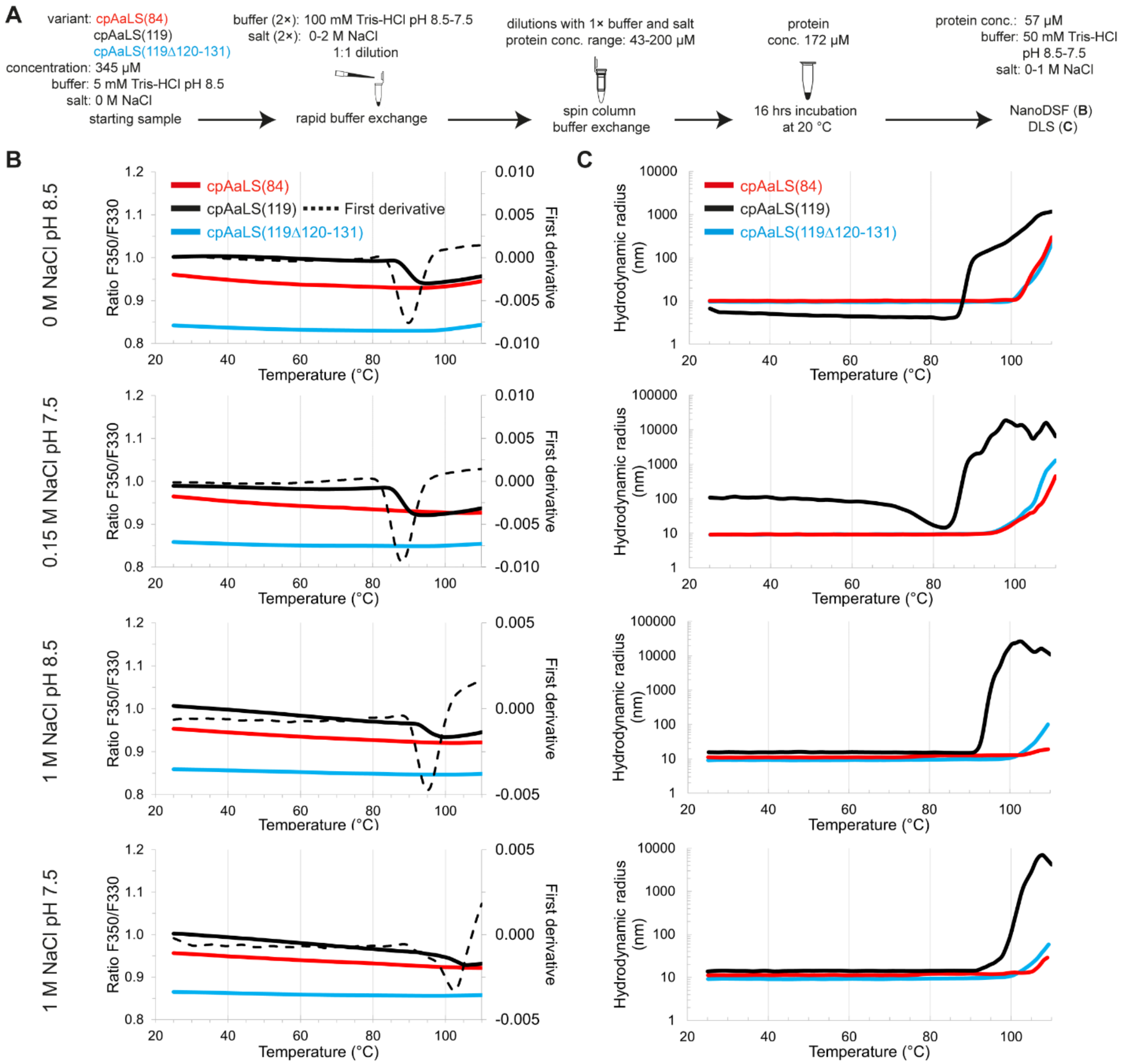

Figure S4. Thermal stability of cpAaLS assemblies.

(A) Experimental scheme for preparation of cpAaLS(84) (red), cpAaLS(119) (black), and cpAaLS(119Δ120-131) (blue) samples in a varied NaCl concentration (0, 0.15, or 1 M) and pH (8.5, or 7.5). (B) Nano-differential scanning fluorimetry (NanoDSF) thermographs of these samples, in the temperature ramp 25–110 ºC. The melting temperature (T_m) of cpAaLS(119) was estimated by the first derivative (dashed lines) minima of the observed fluorescent ratio at F350/F330 to be 90, 88, 96, and 103 °C in the buffers where the major assembly status of the protein should be capsomers, tubular, 24-nm, and 28-nm cages, respectively. (C) Dynamic light scattering of the corresponding samples. Increasing hydrodynamic radii in the temperature ramp indicate aggregation of cpAaLS(119), ahead of cpAaLS(84) or cpAaLS(119Δ120-131).

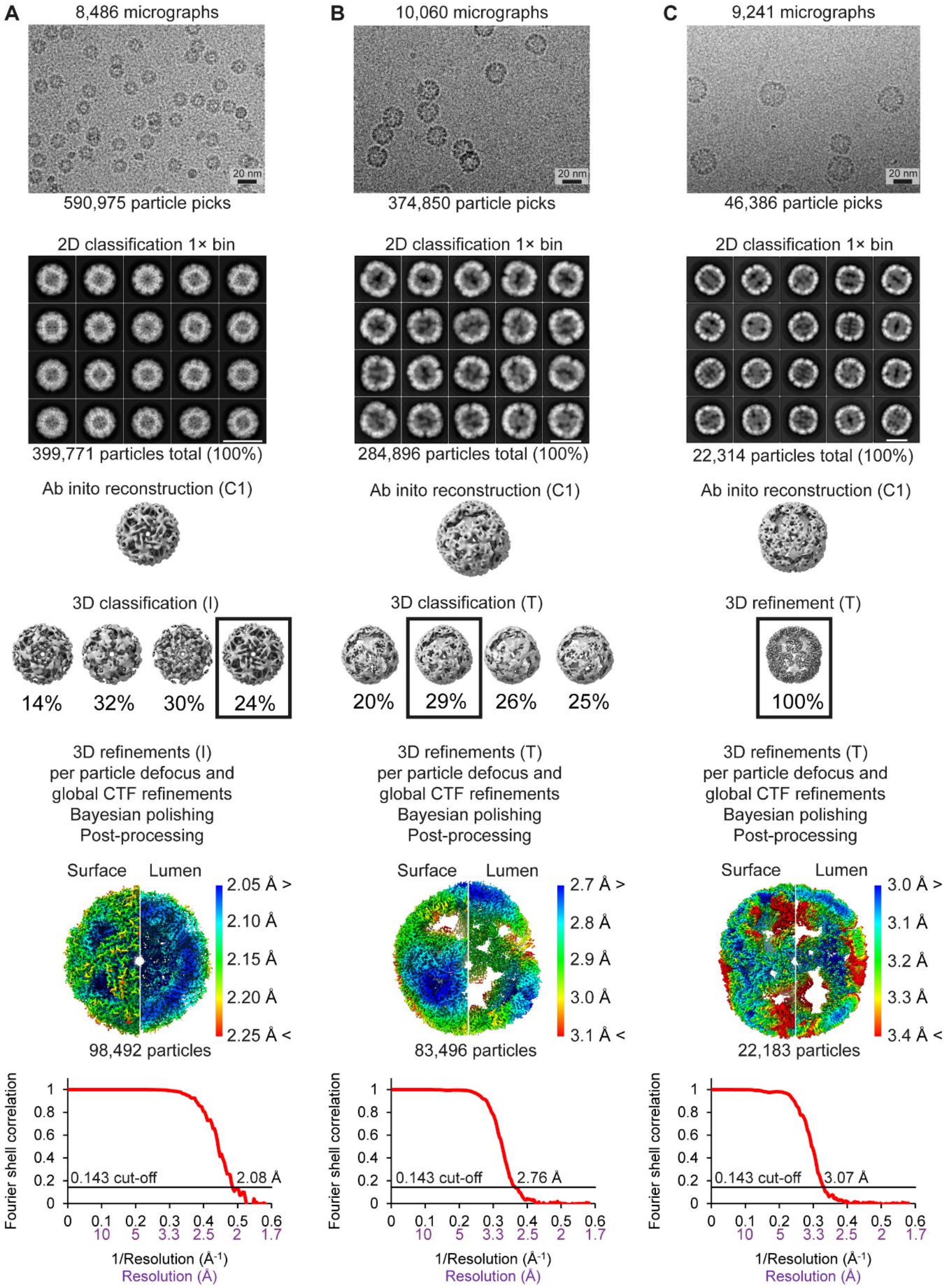
A
8,486 micrographs
20 nm
590,975 particle picks
2D classification 1× bin
399,771 particles total (100%)
Ab inito reconstruction (C1)
3D classification (I)
14%
32%
30%
24%
3D refinements (I)
per particle defocus and
global CTF refinements
Bayesian polishing
Post-processing
Surface
Lumen
2.05 Å >
2.10 Å
2.15 Å
2.20 Å
2.25 Å <
98,492 particles
Fourier shell correlation
0.143 cut-off
2.08 Å
1/Resolution (Å-1)
Resolution (Å)
B
10,060 micrographs
20 nm
374,850 particle picks
2D classification 1× bin
284,896 particles total (100%)
Ab inito reconstruction (C1)
3D classification (T)
20%
29%
26%
25%
3D refinements (T)
per particle defocus and
global CTF refinements
Bayesian polishing
Post-processing
Surface
Lumen
2.7 Å >
2.8 Å
2.9 Å
3.0 Å
3.1 Å <
83,496 particles
Fourier shell correlation
0.143 cut-off
2.76 Å
1/Resolution (Å-1)
Resolution (Å)
C
9,241 micrographs
20 nm
46,386 particle picks
2D classification 1× bin
22,314 particles total (100%)
Ab inito reconstruction (C1)
3D refinement (T)
100%
3D refinements (T)
per particle defocus and
global CTF refinements
Bayesian polishing
Post-processing
Surface
Lumen
3.0 Å >
3.1 Å
3.2 Å
3.3 Å
3.4 Å <
22,183 particles
Fourier shell correlation
0.143 cut-off
3.07 Å
1/Resolution (Å-1)
Resolution (Å)

Figure S5. Single particle reconstruction of cpAaLS(84) and cpAaLS(119) spherical cages.

(A-C) The scheme represents the processing of cryo-EM datasets for the icosahedral (I) cpAaLS(84) 12-pentamer cage (A), tetrahedral (T) cpAaLS(119) 24- (B), or 36-pentamer (C) cages. From top to bottom, the scheme shows the representative micrographs (20 nm black scale bar), selected 2D classes (white scale bar), ab initio reconstruction, 3D classification with selected classes (boxed with black line), and lists per particle/micrograph/movie corrections. Following final 3D cryo-EM refinements, the maps were filtered and colored by local resolution (0.143 FSC cut-off). Gold-standard Fourier shell correlation curves are shown (bottom). The 3D maps are not to scale. The absolute and/or relative (% of total) number of particles is indicated after each selection step.

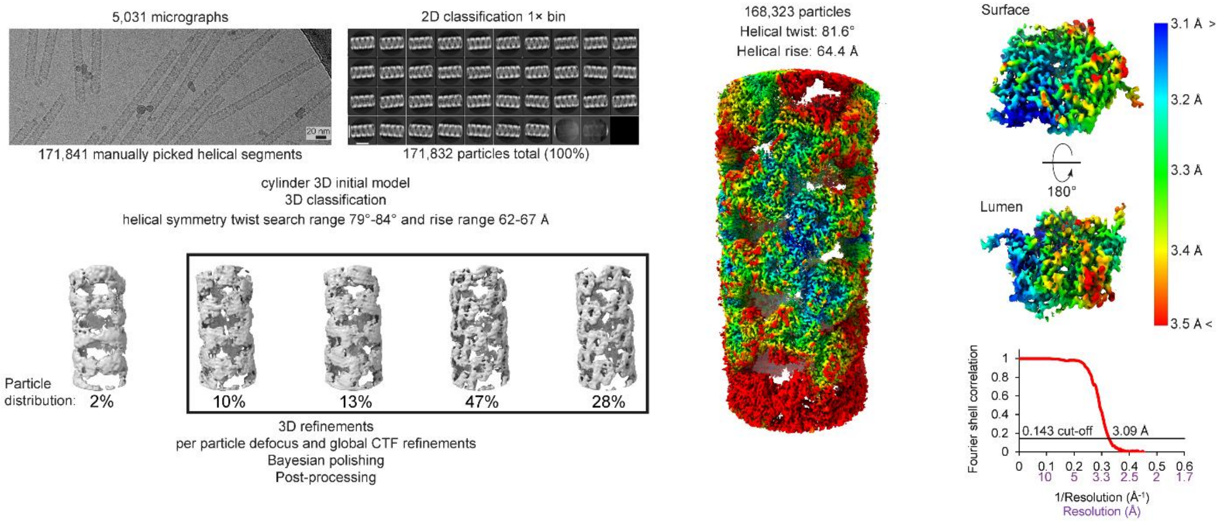

Figure S6. Helical reconstruction of cpAaLS(119) straight tube.

The processing scheme of the cryo-EM dataset shows a representative micrograph, selected 2D classes (white scale bar = 20 nm), 3D classification with selected classes (black box), and per particle/micrograph/movie corrections. Following final 3D cryo-EM refinements the map was filtered and colored by local resolution (0.143 FSC cut-off). The cryo-EM density corresponding to the asymmetric pentamer in the middle of the tubular segment was extracted to display the best local resolution on the surface and in the lumen of the cage. Gold-standard Fourier shell correlation curve was calculated with a 30% helical axis mask (right bottom). The 3D maps are not to scale. The absolute and/or relative (% of total) number of particles is indicated after each selection step.

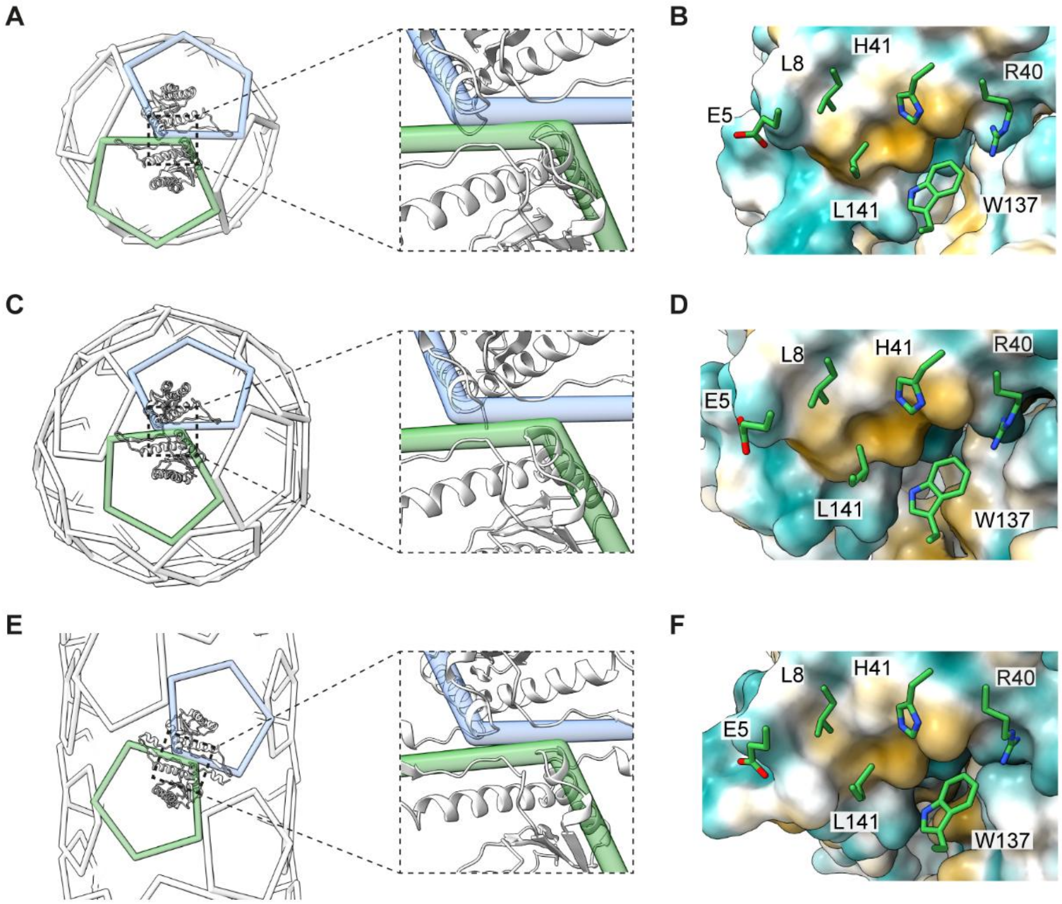

Figure S7. Bonding network between pentamers at the (pseudo) 2-fold symmetry in cpAaLS cages. (A, C, E) Wire diagram of the 12-pentamer cpAaLS (84) (A), 24-pentamer cpAaLS(119) (C) spherical cages, and cpAaLS(119) straight tube assembly (E) with an enlarged view of a representative pentamer pair (green and blue), and cartoon representation of interfacing monomers. (B, D, F) The corresponding surfaces presenting hydrophobic (brown) and hydrophilic (blue) properties. The contacting residues from the opposite monomer are displayed as green sticks. Aside from the varied orientation of these side chains, all the assemblies preserve essentially the same amino acid interaction patterns at the (pseudo) 2-fold symmetrical interface.

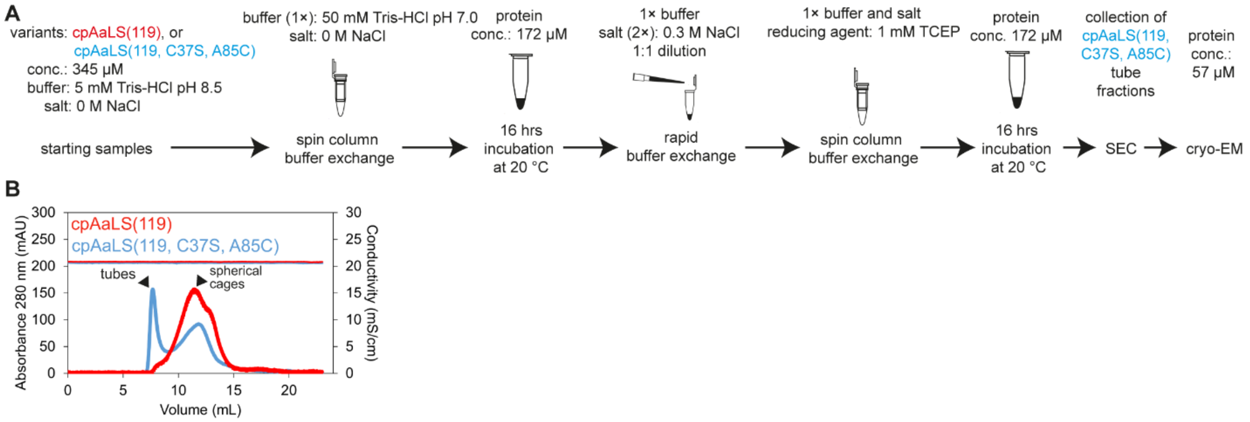

Figure S8. Assembly of cpAaLS(119, C37S, A85C) into twisted tubes.

(A) Experimental scheme for preparation of the twisted tubes. (B) Size-exclusion chromatogram of the cpAaLS(119, C37S, A85C) sample (blue), showing a mixture of twisted tubes and spherical cages. The parent cpAaLS(119) variant forms spherical cages (red) almost exclusively under the same condition. Difference in the assembly states of cpAaLS(119) with 0.15 M NaCl from the results shown in Supplementary Fig S2C might be attributed to the incubation of the protein at pH 7.0, prior to addition of salt.

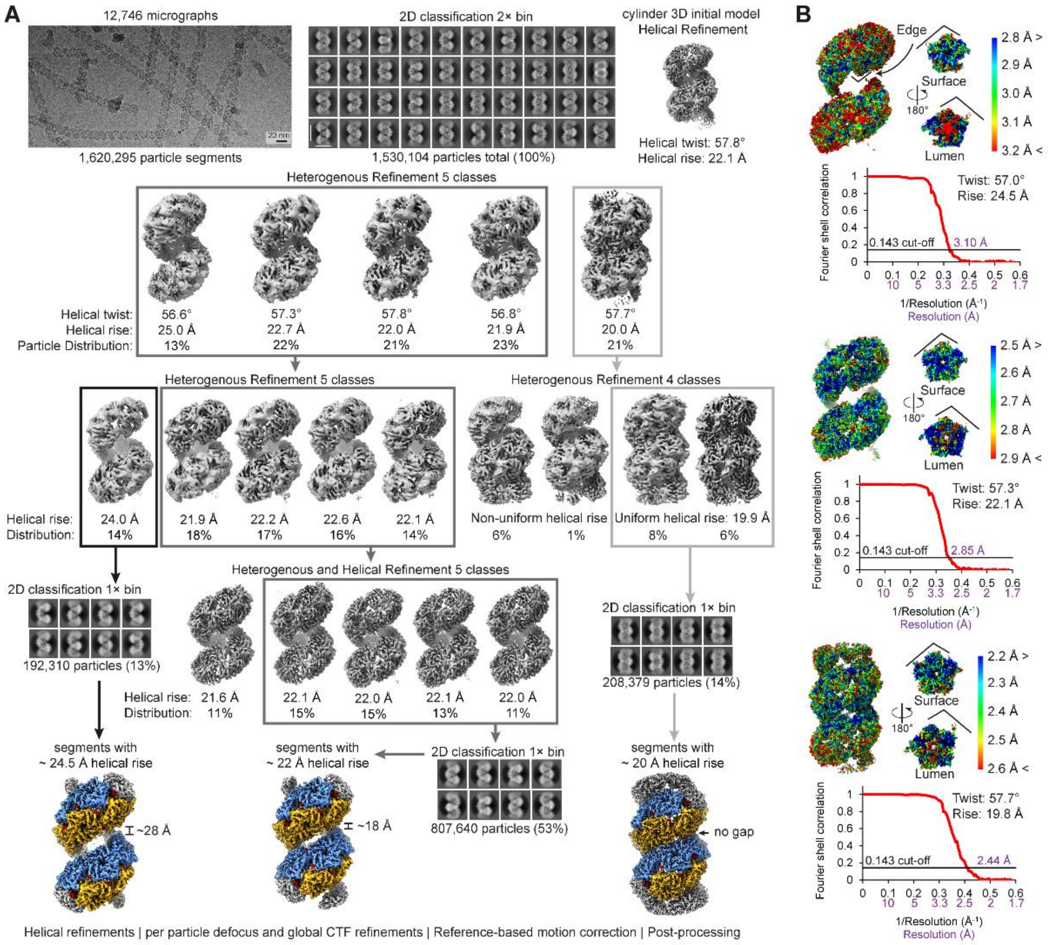

Figure S9. Helical reconstruction of cpAaLS(119, C37S, A85C) twisted tube.

(A) Processing of the cryo-EM dataset showing the representative micrograph (20 nm black scale bar), selected 2D classes (20 nm white scale bar), and initial helical refinement. The non-uniform helical segments (particles) were separated based on the helical rise parameter. The segments with ~24.5-, ~22-, or ~20-Å helical rise were obtained by iterative 3D heterogeneous refinements (selected 3D classes are boxed with black, dark grey, and light grey lines, respectively), and confirmed by additional 2D classification. The final particle stacks were used to obtain uniform structures with ~28-Å, ~18-Å, or no gap (0-Å) between dual helical strips, respectively (orange/blue strips, bottom left, middle and right). The absolute and/or relative (% of total) number of particles is indicated after each selection step. (B) Following per particle/micrograph/movie corrections and final 3D cryo-EM refinements, the corresponding maps were filtered and colored by local resolution (0.143 FSC cut-off). The cryo-EM densities corresponding to representative asymmetric pentamers were extracted to display the local resolution on the surface and in the lumen of the cages. The edge of the pentamers exposed to the gap between helical strips is indicated. Gold-standard Fourier shell correlation curve is shown. The 3D maps are not to scale.

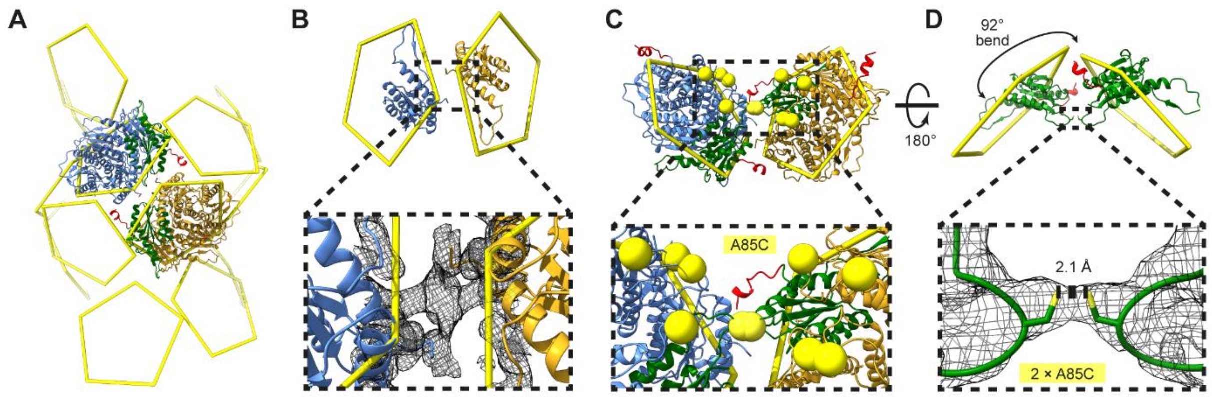

Figure S10. Potential disulfide bridge facilitating the acute bending angle in the pentamer-pentamer interaction.

Potential disulfide bridge facilitating the acute bending angle in the pentamer-pentamer interaction. (A) The wire (yellow) representation of cpAaLS(119, C37S, A85C) twisted tube with no gap (0-Å gap) between dual strips. A pentamer pair interacting via acute bending angle is shown with ribbon representation. (B) Amplified view of the acute interaction between the adjacent protomers (blue and golden ribbons), indicating that this is not held by the hydrophobic (L8, L141, W137) or hydrophilic (E5, H41, R40) amino acids, unlike other pentamer-pentamer contacts exemplified in Figure S2B, D, and F. The unfitted cryo-EM density (expanded region, black mesh) may correspond to the flexible 'untethered' α-helix(120-131). (C) Extracted pentamer pair shown from the lumen with A85C cysteines highlighted as yellow spheres. Two of the cysteines are in proximity (expanded region, green protomers). (D) The pentamer pair with isolated protomers (green ribbon) shown from the side. The continuous cryo-EM density (expanded region, black mesh) and optimal distance (2.1 Å) between the proximal A85C residues are suggestive of disulfide bond formation despite the presence of 1 mM TCEP. Similarly, close proximity of the A85C residues was also observed in 18- and 28-Å gap counterparts (not shown). Models and maps are not to scale.

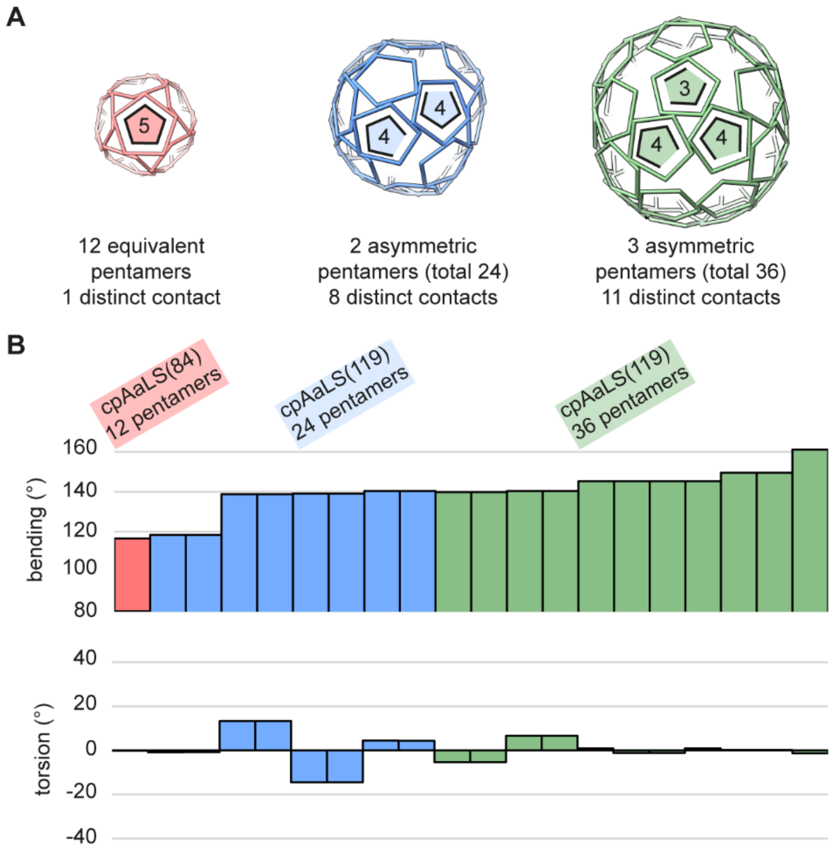

Figure S11. Capsomer interaction angles in spherical cages.

(A) The wire representation of 12-pentamer cpAaLS(84) (red), 24-pentamer cpAaLS(119) (blue), and 36-pentamer cpAaLS(119) (green) assemblies. The numbers of contacts per each asymmetric pentamer are indicated. (B) Measured values of the bending (top) and the torsion angles (bottom) between two pentamers.

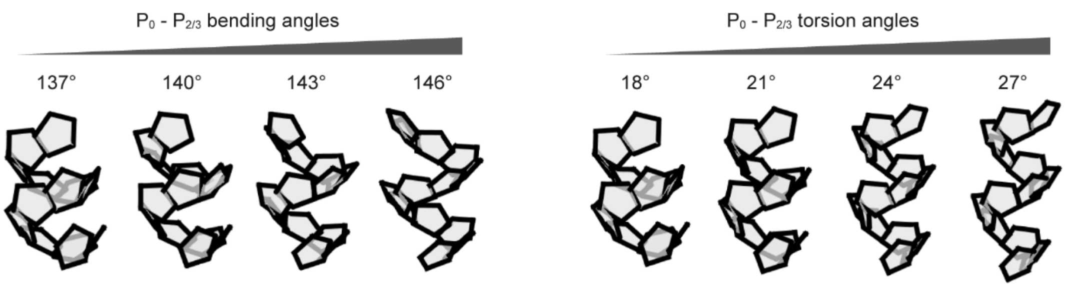

Figure S12. Correlation between pentamer interaction angles and helical rise.

Computational simulation using a pentagon-based helical model showing that increasing bending (left) or torsion angle (right) for the P_0-$P_{2/3}$ pentamer interface (Figure S4D) in twisted tubes results in the widening gap between helical strips. The simulation results agree with the experimental observations shown in Figure S4, B to F.

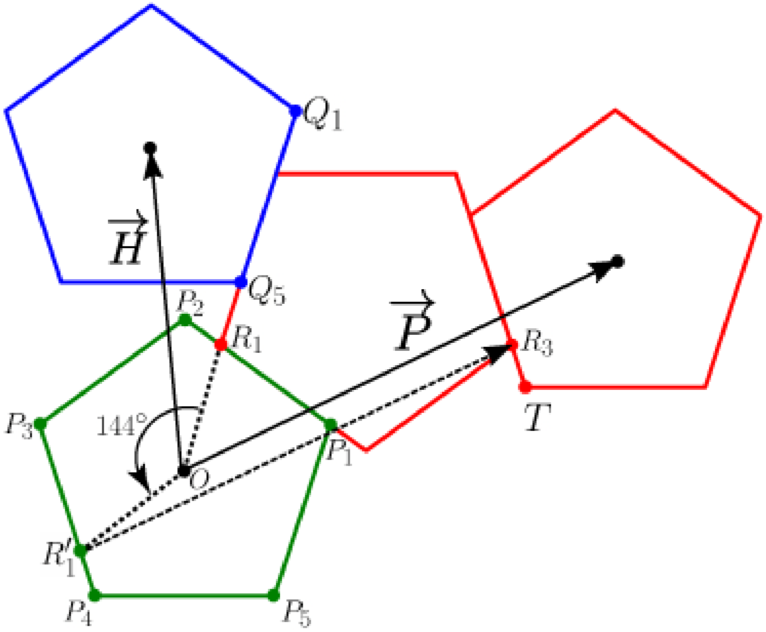

Figure S13. Mathematical characterization of the straight tubes.

Taking the center of the green pentagon as the origin (O), vectors $\vec{H}$ and $\vec{P}$ specify the helicity and periodicity directions, respectively. Since the two adjacent green and red pentagons are translated as a unit, the origin is mapped to the center of the second pentagon (black dots). Labels refer to the variables used in the proof (Materials and Methods, Mathematical characterization of the straight tubes).

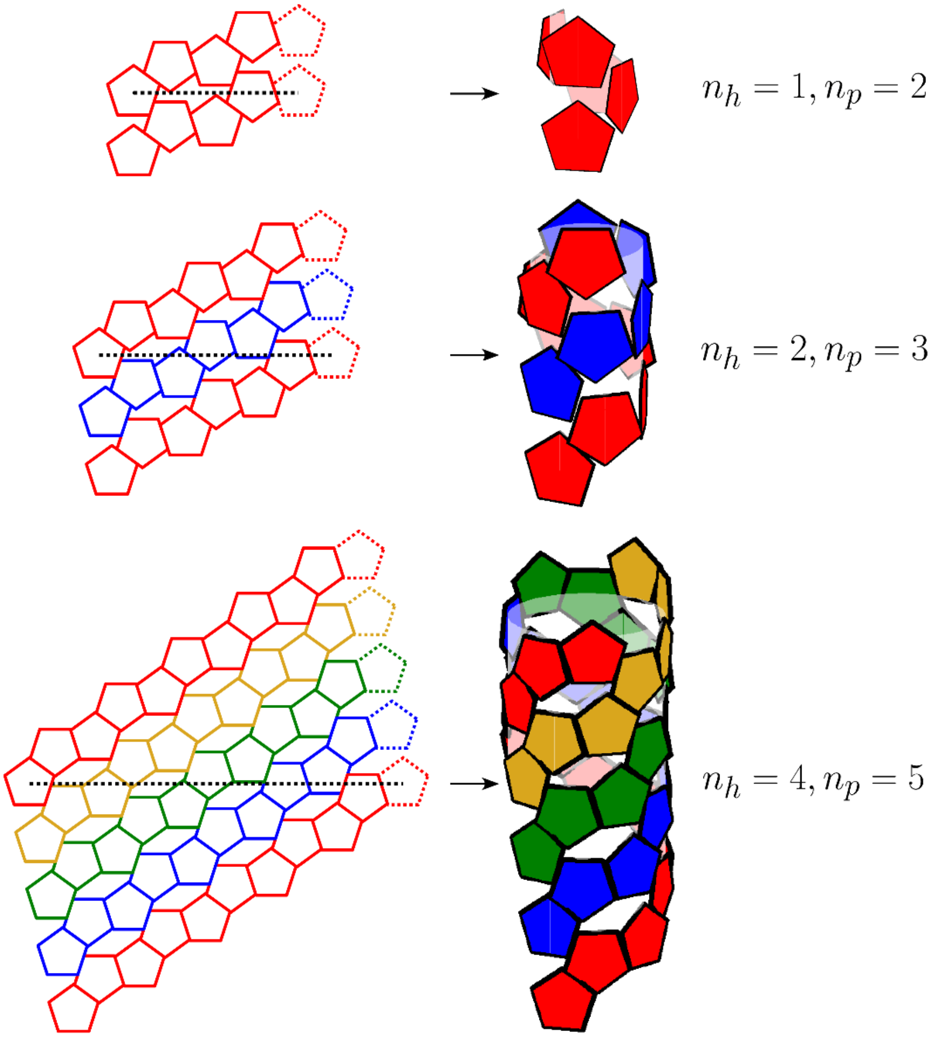

Figure S14. Tiling pattern and resulting tubular structures.

Three geometrically possible tilling patterns with various n_h and n_p numbers and the corresponding 3D tube models are shown as examples. Individual pentamer threads are shown in different colors. The pentamers connected with the black dashed lines are identical in the 3D tubular structure.

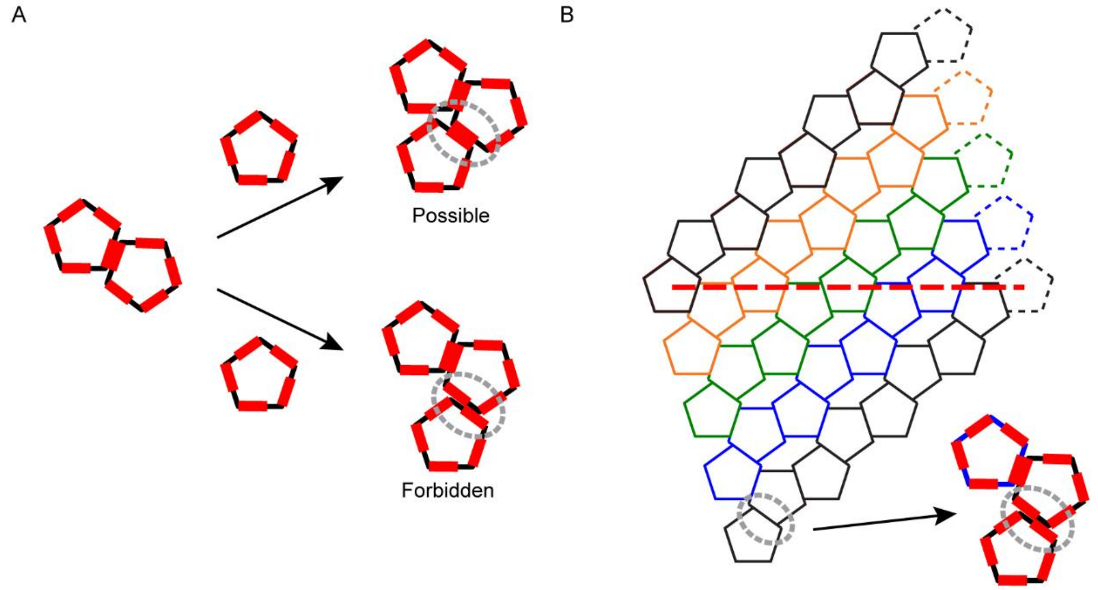

Figure S15. Tiling pattern violating the biologically occurring interactions.

(A) Schematic representation of possible and forbidden interaction patterns for three pentamers having an asymmetric interaction surface (presented as red patches). (B) Example of a geometrically possible but biologically forbidden tilling pattern. This tilling pattern, defined by (n_h, n_p) = (4, 4), requires flipped pentagons to retain the same type of interfaces, which is unlikely to occur with protein building blocks. The pentamers connected with the red dashed line are identical in the 3D tubular structure.

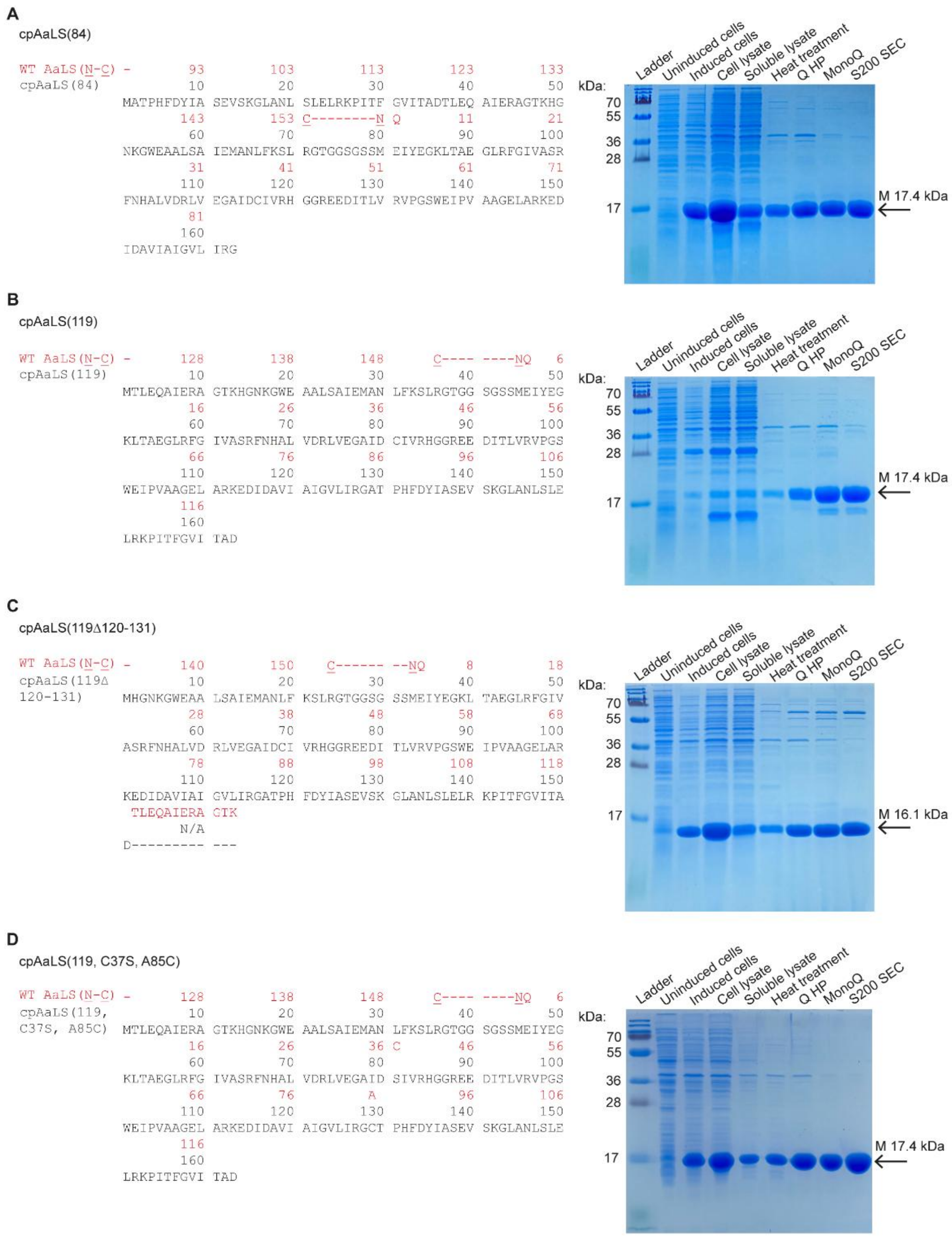

Figure S16. Primary sequence and purification of cpAaLS variants.

(A-D) Primary sequence (left) and SDS-PAGE analysis (right) of cpAaLS(84) (A), cpAaLS(119) (B), cpAaLS(119Δ120-131) (C), and cpAaLS(119, C37S, A85C) (D) variants. Each primary sequence (in black) is aligned to wild-type AaLS (in red) showing its native termini (N-C), gaps (-), amino acid substitutions (i.e. C37C), or truncations (Δ120-131). The arrows next to the respective gels indicate the band corresponding to the molecular mass (M in kDa) calculated from the amino acid sequence using the Expasy ProtParam tool.

Table S1. Oligonucleotides used in this study.

Name	Sequence
FW_NdeI_cpAaLS(119Δ120-131)	CATCATATGCACGGCAACAAAGGTTGG
RV_XhoI_cpAaLS(119Δ120-131)	CATCATCTCGAGTTAGTCAGCTGTAATAAC
FW_cpAaLS(119, C37S)	CGTCTGGTGGAGGGTGCAATTGATaGCATAGTCCGTCATGGCGGC
RV_cpAaLS(119, C37S)	GCCGCCATGACGGACTATGCtATCAATTGCACCCTCCACCAGACG
FW_cpAaLS(119, C37S, A85C)	CAATTGGCGTTCTCATCAGAGGCTGCACGCCACATTTCGATTATATCGCC
RV_cpAaLS(119, C37S, A85C)	GGCGATATAATCGAAATGTGGCGTGCAGCCTCTGATGAGAACGCCAATTG

Table S2. Plasmids used in this study.

Name	Gene	Promoter/Operator[a]	Ori	Marker[b]	Ref.
pMG_cpAaLS_L8 (119)	cpAaLS (119)	P_{T7} / *lac*O	pBR322	Amp^R	39
pMG_cpAaLS_L8 (84)	cpAaLS (84)	P_{T7} / *lac*O	pBR322	Amp^R	41
pMG_cpAaLS_L8 (119Δ120-131)	cpAaLS (119Δ120-131)	P_{T7} / *lac*O	pBR322	Amp^R	This study
pMG_cpAaLS_L8 (119, C37S)	cpAaLS (119, C37S)	P_{T7} / *lac*O	pBR322	Amp^R	This study
pMG_cpAaLS_L8 (119, C37S, A85C)	cpAaLS (119, C37S, A85C)	P_{T7} / *lac*O	pBR322	Amp^R	This study

[a] P_{T7} / *lac*O, T7 promoter combined with lactose operator.

[b] Amp^R, ampicillin resistance.

Table S3. Cryo-EM data collection and structure refinement statistics.

**Variant**	cpAaLS(84)	cpAaLS(119)	cpAaLS(119)	cpAaLS(119)	cpAaLS(119, C37S, A85C)		
**Assembly**	12-pentamer spherical cage	24-pentamer spherical cage	36-pentamer spherical cage	Straight tube	Twisted tube 28-Å gap	Twisted tube 18-Å gap	Twisted tube 0-Å gap
**EMDB**	51006	51005	51004	51003	51001	51000	50999
**PDB**	9G3P	9G3O	9G3N	9G3M	9G3J	9G3I	9G3H
**Data collection and processing**							
Magnification	105,000 ×	105,000 ×	105,000 ×	81,000 ×	105,000 ×	105,000 ×	105,000 ×
Voltage (keV)	300	300	300	300	300	300	300
Electron exposure ($e^-/Å^2$)	40	40	40	40	42.44	42.44	42.44
Defocus range	-0.9 to -2.1	-0.9 to -2.1	-0.9 to -2.1	-0.9 to -3.0	-0.9 to -1.5	-0.9 to -1.5	-0.9 to -1.5
Pixel size (Å)	0.86	0.86	0.86	1.1[a]	0.846	0.846	0.846
Micrographs (no.)	8,486	10,060	9,241	5,031	12,746	12,746	12,746
Symmetry imposed	I	T	T	81.6 º twist 64.4 Å rise	57.0 º twist 24.5 Å rise	57.3 º twist 22.1 Å rise	57.7 º twist 19.8 Å rise
Initial particle images (no.)	590,975	374,850	46,386	171,841	1,620,295	1,620,295	1,620,295
Final particle images (no.)	98,492	83,496	22,183	168,323	192,310	807,640	208,379
Map resolution (Å)	2.08	2.76	3.07	3.09	3.10	2.85	2.44
FSC threshold	0.143	0.143	0.143	0.143	0.143	0.143	0.143
Map resolution range (Å) 25th–75th percentile	2.0–2.3	2.7–3.2	3.0–4.0	3.0–4.0	2.9–4.5	2.6–3.7	2.3–3.2
Map sharpening B-Factor	65.0	99.3	87.9	107.1	102.5	115.2	79.6
**Model building**							
PDB code of the initial model: 1HQK							
Model composition							
Chains	60	120	180	150	100	100	100
Protein residues	9600	17856	26652	23010	15260	15260	15260
Non-hydrogen atoms	72480	133824	199560	172740	114640	114640	114640
Water/Ligands	0	0	0	0	0	0	0
Nucleotides	0	0	0	0	0	0	0
B factors (mean $Å^2$)	32.62	50.76	96.35	74.82	118.52	110.28	78.30
R.M.S. deviations							
Bond lengths (Å)	0.004	0.005	0.004	0.004	0.004	0.004	0.005
Bond angles (º)	0.963	0.974	0.943	0.945	0.926	0.915	0.994
Validation							
MolProbity score	0.98	1.17	1.22	1.07	1.22	1.18	1.21
Clash score	2.10	1.69	2.62	1.87	2.56	3.60	2.79
Poor rotamers (%)	0.11	0.00	0.06	0.00	0.00	0.00	0.00
Ramachandran							
Favored (%)	98.10	96.28	97.04	97.38	96.95	97.88	97.21
Allowed (%)	1.90	3.72	2.96	2.62	3.05	2.12	2.79
Outliers (%)	0.00	0.00	0.00	0.00	0.00	0.00	0.00
CC (volume)	0.86	0.78	0.77	0.76	0.86	0.86	0.90

[a] Super-resolution pixel size 0.55 Å
